# Supplementary material for: Detailed Speciation of Non-Methane Volatile Organic Compounds in Exhaust Emissions from Diesel and Gasoline Euro 5 Vehicles Using Online and Offline Measurements
Source: Toxics. 2022 Apr 8;10(4):184. doi: 10.3390/toxics10040184 (PMC9032894; doi:10.3390/toxics10040184)
Supplement: Supplementary file 1 [file toxics-10-00184-s001.zip › toxics-1639238-supplementary.pdf]

toxics

MDPI

Supplementary Materials: Detailed Speciation of Non-Methane Volatile Organic Compounds in Exhaust Emissions from Diesel and Gasoline Euro 5 Vehicles Using Online and Offline Measurements

Baptiste Marques, Evangelia Kostenidou, Alvaro Martinez Valiente, Boris Vansevenant, Thibaud Sarica, Ludovic Fine, Brice Temime-Roussel, Patrick Tassel, Pascal Perret, Yao Liu, Karine Sartelet, Corinne Ferronato, and Barbara D'Anna

1. PMF Analysis

1.1. Diesel Cold Urban Cycle

Preliminary tests were carried on the diesel cold urban cycle dataset. Different input formats were investigated in order to better describe the data. The first PMF tests were done considering all the ions signals from the peak list used to treat the PTR-ToF-MS raw data, sampled with a temporal resolution of 1 second during 4 cycles of 994 seconds for a total time of 3976 seconds. Tests from 1 to 10 factors were run to cover a wide range of possible solutions, with the appliance of a step function to downweight the impact of variables with S/N (signal to noise ratio) under 2. Q/Qexp values in this configuration ranged from 4.7 for the solution with 1 factor to 1.22 for the solution with 10 factors. The passing from 1 to 2 factors and from 3 to 4 factors induced high decreases in the Q/Qexp values and increases in the absolute total concentration described by the factors, signifying that solutions with 4 factors or more were better at explaining the data variations. Solutions with 7 to 10 factors exhibited abnormally high calculated versus experimental absolute total concentration values and were therefore excluded as they did not seem to describe well the data. The passage from 4 to 5 factors and from 5 to 6 factors exhibited no significant evolution in the runs characteristics, whether it be on the Q/Qexp value or the absolute calculated concentration. Visual evaluation of the temporal variations of the different factors from these solutions highlighted that solutions with 5 and 6 factors underwent factor splitting. We thus chose to focus on the solution with 4 factors that seemed to be the most meaningful solution in respect with the driving conditions and the aftertreatment systems functioning. Despite the apparent physical validity of the solution, different parameters convinced us to deepen our analysis. First of all, although the scaled residuals histogram showed an unimodal gaussian-like distribution, meaning that the solution does not present any systematic under- or overestimation, the ratio of unexplained variations (UEV) reached 42%, well above the maximum 25% required by a variable to be regarded as explained. Secondly, we noticed that variables with S/N (signal to noise ratio) under 2 were mainly constituted of noise, resulting in failed attempts to describe them with the different factors and explaining the high proportion of the "noisy" UEV compared to the "real" UEV. It appeared that compounds with S/N ratios under 2 all presented the same factor to specie ratios, with values of 0.15, 0, 0.7 and 0.15 for the factors 1, 2, 3 and 4 respectively. We reconstituted the total signal from variables with S/N under 2 based on their factor to specie ratios. It poorly described the total variations of the variables, but fitted well within the large error bars, confirming that artifacts from noisy variables were falsely attributed to real variations. To avoid this, we chose to filter the input data matrix from all the variables with S/N under 2 and redo the PMF analysis. Following the same choices as for the unfiltered data, the solution with 4 factors was selected as the most descriptive of the driving conditions and vehicle characteristics. This solution is in fact almost the same as precedingly since the variables with S/N under 2 were already downweighed. The main evolution concerned the proportion of UEV that decreased from 42%

to 32% in this configuration. Although this value is still above 25%, the solutions were considered trustful. The high UEV in both configurations were attributed to the specificities of the experiments. From now on, all the PMF inputs were filtered from all the variables with S/N under 2.

1.2. Diesel Motorway Cycle

PMF tests for the diesel motorway cycle were done considering all the ions signals with S/N above 2, sampled with a temporal resolution of 1 second during 2 cycles of 1068 seconds for a total time of 2136 seconds. Tests from 1 to 10 factors were run to cover a wide range of possible solutions. Q/Qexp values in this configuration ranged from 8.4 for the solution with 1 factor to 2.1 for the solution with 10 factors. The passing from 1 to 2, from 2 to 3 and from 3 to 4 factors induced high decreases in the Q/Qexp values and increases in the absolute total concentration described by the factors, signifying that solutions with 4 factors or more were better at explaining the data variations. The 10 factors solution exhibited higher calculated versus experimental absolute total concentration values and was therefore excluded as it did not seem to describe well the data. Solutions with 5 to 9 factors exhibited a decrease in their Q/Qexp values, but no increase in the absolute total concentration described by the factors. Moreover, solutions from 6 to 9 factors underwent factor splitting. We thus chose the 5 factors solution as the most meaningful solution, with a satisfying UEV of 25%, a calculated versus absolute total concentration ratio of 100%, and an unimodal Gaussian-like distribution of the scaled residuals.

1.3. GDI Cold Urban Cycle

PMF tests for the GDI cold urban cycle were done considering all the ions signals with S/N above 2, sampled with a temporal resolution of 1 second during 6 cycles of 994 seconds for a total time of 5964 seconds. Tests from 1 to 10 factors were run to cover a wide range of possible solutions. Q/Qexp values in this configuration ranged from 13.5 for the solution with 1 factor to 4.1 for the solution with 10 factors. The passing from 1 to 2 factors induced high decreases in the Q/Qexp values. Moreover, passing from 1 to 2 and from 4 to 5 factors induced clear increases in the absolute total concentration described by the factors, signifying that solutions with 5 factors or more were better at explaining the data variations. Passing from 5 to 6 factors induced a high decrease from 40 to 29% of the UEV. Solutions with 7 to 10 factors exhibited relatively low decreases in their Q/Qexp values, and no increase in the absolute total concentration described by the factors. Moreover, solutions from 7 to 10 factors underwent factor splitting. We thus chose the 6 factors solution as the most meaningful solution, with an UEV of 29%, a calculated versus absolute total concentration ratio of 97%, and an unimodal Gaussian-like distribution of the scaled residuals.

1.4. PFI Cold Urban Cycle

PMF tests for the PFI cold urban cycle were done considering all the ions signals with S/N above 2, sampled with a temporal resolution of 1 second during 1 cycles of 994. However, due to the high concentrations of BTEX and small alkenes leading to the saturation of these signals during the cold start of the vehicle, all ions at *m/z* 45, 57, 93, 107 and 121 were removed from the analysis. Tests from 1 to 10 factors were run to cover a wide range of possible solutions. Q/Qexp values in this configuration ranged from 3.9 for the solution with 1 factor to 1.6 for the solution with 10 factors. The passing from 1 to 2 and 3 to 4 factors induced high decreases in the Q/Qexp values. Moreover, passing from 1 to 4 factors induced clear increases in the absolute total concentration described by the factors, signifying that solutions with 4 factors or more were better at explaining the data variations. 7 to 10 factors solutions exhibited higher calculated versus experimental absolute total concentration values and were therefore excluded as they did not seem to describe

well the data. The solution with 4, 5 and 6 factors exhibits similar absolute total concentration values, with a slightly higher value for the 5 factors solution. Moreover, solutions with 6 factors underwent factor splitting. We thus chose the 5 factors solution as the most meaningful solution, with an UEV of 24%. Results for this cycle are presented in figure S2.

Table S1. Experimental schedule and dilutions.

Year	Vehicle type	Date	cycle type	PTR-ToF-MS	GC-MS	CVS	Ejector dilution	Clean air dilution	Fast GC dilution	PTR-ToF-MS total dilution	GC-MS total dilution
2018	Diesel	12/04/2018	Urban cold	YES	-	-	15	1	1	15	-
			Motorway	YES	-	-	15	1	1	15	-
			Motorway	YES	-	-	15	1	1	15	-
		16/04/2018	Urban cold	-	-	YES	-	-	-	-	-
			Motorway	-	-	YES	-	-	-	-	-
		18/04/2018	Urban cold	-	-	YES	-	-	-	-	-
			Motorway	-	-	YES	-	-	-	-	-
		20/04/2018	Motorway	-	YES	-	2.3	-	-	-	2.3
		23/04/2018	Urban cold	YES	-	YES	2.3	3.4	1	7.8	-
		24/04/2018	Urban cold	YES	-	-	2.3	3.4	1	7.8	-
		25/04/2018	Urban cold	YES	-	-	-	-	-	-	-
			Motorway	-	YES	-	-	-	-	-	2.3
			Motorway	-	YES	-	2.3	3.4	1	7.8	2.3
	Gasoline PFI	15/05/2018	Motorway	YES	-	-	2.3	3.4	1	7.8	-
			Motorway	YES	-	-	2.3	3.4	1	7.8	-
			Motorway	YES	-	-	2.3	3.4	1	7.8	-
		23/05/2018	Motorway	-	YES	-	2.3	-	-	-	2.3
			Motorway	-	YES	-	2.3	-	-	-	2.3
			Motorway	-	YES	-	2.3	-	-	-	2.3
		25/05/2018	Urban cold	-	-	YES	-	-	-	-	-
			Motorway	YES	-	YES	2.3	3.7	1	8.4	-
			Motorway	YES	-	-	2.3	3.7	1	8.4	-
		28/05/2018	Urban cold	YES	-	-	2.3	3.7	1	8.4	-
2019	Gasoline DI	04/04/2019	Urban cold	YES	-	YES	1.5	6.6	2.5	23.4	-
			Motorway	YES	-	YES	1.5	5.2	2.5	18.5	-
			Motorway	YES	-	YES	1.5	5.2	2.5	18.5	-
		05/04/2019	Urban cold	YES	-	YES	1.5	6.6	2.5	23.4	-
			Motorway	YES	-	YES	1.5	5.2	2.5	18.5	-
			Motorway	YES	-	YES	1.5	5.2	2.5	18.5	-
		09/04/2019	Urban cold	YES	-	YES	1.5	6.6	2.5	23.4	-
			Motorway	YES	-	YES	1.5	5.2	2.5	18.5	-
			Motorway	YES	-	YES	1.5	5.2	2.5	18.5	-
		11/04/2019	Urban cold	YES	-	YES	1.5	6.6	2.5	23.4	-
			Motorway	YES	-	YES	1.5	5.2	2.5	18.5	-
			Motorway	YES	-	YES	1.5	5.2	2.5	18.5	-
		12/04/2019	Urban cold	YES	-	YES	1.5	6.6	2.5	23.4	-
			Motorway	YES	-	YES	1.5	5.2	2.5	18.5	-
			Motorway	YES	-	YES	1.5	5.2	2.5	18.5	-
		16/04/2019	Urban cold	YES	-	YES	1.5	6.6	2.5	23.4	-
			Motorway	YES	-	YES	1.5	5.2	2.5	18.5	-
			Motorway	YES	-	YES	1.5	5.2	2.5	18.5	-
		18/04/2019	Motorway	YES	-	YES	1.5	5.2	2.5	18.5	-
			Motorway	YES	-	YES	1.5	5.2	2.5	18.5	-
		19/04/2019	Urban cold	-	YES	-	8.4	-	-	-	8.4
		23/04/2019	Urban cold	-	YES	-	8.4	-	-	-	8.4

2019	Gasoline DI	23/04/2019	Motorway	-	YES	-	8.4	-	-	-	8.4
		24/04/2019	Urban cold	-	YES	-	8.4	-	-	-	8.4
			Motorway	-	YES	-	8.4	-	-	-	8.4
		26/04/2019	Urban cold	-	YES	-	8.4	-	-	-	8.4
			Motorway	-	YES	-	8.4	-	-	-	8.4

Table S2. Factor to species contribution ratios for the diesel cold urban cycle factor 1.

ion family	Ion formula	factor 1 contribution to the signal (%)	Attribution
$C_nH_{2n}^+$	$C_2H_4^+$	36	Ethene
$(C_nH_{2n})H^+$	$(C_3H_6)H^+$	96	Propene
	$(C_4H_8)H^+$	58	C4 alkenes
	$(C_5H_{10})H^+$	22	C5 alkenes
	$(C_6H_{12})H^+$	5	C6 alkenes/Cyclohexane
$(C_{n+2}H_{2n+2})H^+$	$(C_3H_4)H^+$	88	C4 alkenes fragment
	$(C_4H_6)H^+$	50	Butadiene
	$(C_5H_8)H^+$	19	C5 diene/cyclopentene
	$(C_6H_{10})H^+$	1	C6 diene/cyclohexene
$(C_{n+6}H_{2n+6})H^+$	$(C_6H_6)H^+$	73	Benzene
	$(C_7H_8)H^+$	40	Toluene
	$(C_8H_{10})H^+$	15	C8 monoaromatics
	$(C_9H_{12})H^+$	6	C9 monoaromatics
	$(C_{10}H_{14})H^+$	4	C10 monoaromatics
	$(C_{11}H_{16})H^+$	2	C11 monoaromatics
	$(C_{12}H_{18})H^+$	2	C12 monoaromatics
	$(C_{13}H_{20})H^+$	2	C13 monoaromatics
	$(C_{14}H_{22})H^+$	2	C14 monoaromatics
	$(C_{15}H_{24})H^+$	3	C15 monoaromatics
$(C_{n+9}H_{2n+10})H^+$	$(C_{10}H_{12})H^+$	5	C10 naphthenics monoaromatics
	$(C_{11}H_{14})H^+$	2	C11 naphthenics monoaromatics
	$(C_{12}H_{16})H^+$	7	C12 naphthenics monoaromatics
$(C_nH_{2n+2}O)H^+$	$(CH_3OH)H^+$	83	Methanol
	$(C_2H_5OH)H^+$	53	Ethanol
$(C_nH_{2n}O)H^+$	$(CH_2O)H^+$	91	Formaldehyde
	$(C_2H_4O)H^+$	68	Acetaldehyde
	$(C_3H_6O)H^+$	1	Acetone/propanal
	$(C_4H_8O)H^+$	1	C4 carbonyl
$(C_{n+1}H_{2n}O)H^+$	$(C_3H_4O)H^+$	76	Acrolein
	$(C_4H_6O)H^+$	32	Methacrolein/crotonaldehyde
	$(C_5H_8O)H^+$	8	C5 unsaturated carbonyl
	$(C_6H_{10}O)H^+$	7	C6 unsaturated carbonyl
$(C_{n+3}H_{2n+2})H^+$	$(C_4H_4O)H^+$	97	Furan
	$(C_5H_6O)H^+$	16	Methylfurane/pyran
	$(C_6H_8O)H^+$	8	dimethylfuran/ethylfuran/Cyclohexenone
$(C_{n+5}H_{2n+2})H^+$	$(C_6H_4O)H^+$	5	Epoxybenzene/ethynylfuran/fragment
	$(C_7H_6O)H^+$	2	Benzaldehyde/tropone
	$(C_8H_8O)H^+$	5	methylbenzaldehyde/phenylacetaldehyde/acetophe none
$(C_nH_{2n}O_2)H^+$	$(C_3H_6O_2)H^+$	6	C3 acids
	$(C_4H_8O_2)H^+$	6	C4 acids
$C_{n+1}H_{2n}O_2)H^+$	$(C_2H_2O_2)H^+$	7	Glyoxal
	$(C_3H_4O_2)H^+$	13	Acrylic acid/ methylglyoxal/propanedial
	$(C_4H_6O_2)H^+$	17	C4 dial/dione/unsaturated acid
	$(C_5H_8O_2)H^+$	12	C5 dial/dione/unsaturated acid
$(C_{n+5}H_{2n+4}O_3)H^+$	$(C_6H_6O_3)H^+$	1	Hydroxymethylfurfural/C6 anhydride

$(C_nH_{2n+1}NO_2)H^+$	$(CH_3NO_2)H^+$	16	Nitromethane
	$(C_2H_5NO_2)H^+$	13	Nitroethane
$(C_nH_{n+1}NO_2)H^+$	$(C_2H_3NO_2)H^+$	17	Nitroethylene
$(C_nH_{n+1}N)H^+$	$(C_2H_3N)H^+$	31	Acetonitrile

Table S3. Factor to species contribution ratios for the diesel cold urban cycle factor 2.

ion family	Ion formula	factor 2 contribution to the signal (%)	Attribution
$C_nH_{2n}^+$	$C_2H_4^+$	62	Ethene
$(C_nH_{2n})H^+$	$(C_5H_{10})H^+$	1	C5 alkenes
	$(C_6H_{12})H^+$	1	C6 alkenes/Cyclohexane
	$(C_7H_{14})H^+$	7	Alkanes fragments
	$(C_9H_{18})H^+$	2	C9 alkanes
$(C_{n+6}H_{2n+6})H^+$	$(C_3H_4)H^+$	9	Aldehydes fragment
	$(C_4H_6)H^+$	16	Aldehydes fragment
	$(C_5H_8)H^+$	3	Aldehydes/cycloalkanes fragment
	$(C_6H_{10})H^+$	6	Aldehydes/cycloalkanes fragment
	$(C_7H_{12})H^+$	8	C7 cycloalkanes
	$(C_8H_{14})H^+$	10	C8 cycloalkanes
	$(C_9H_{16})H^+$	7	C9 cycloalkanes
	$(C_{10}H_{18})H^+$	3	C10 cycloalkanes
	$(C_{13}H_{24})H^+$	4	C13 cycloalkanes
$(C_{n+5}H_{2n+7})H^+$	$(C_6H_8)H^+$	8	Bicycloalkanes fragment
	$(C_7H_{10})H^+$	2	Bicycloalkanes fragment
	$(C_8H_{12})H^+$	0	Bicycloalkanes fragment
	$(C_9H_{14})H^+$	4	C9 bicycloalkanes
	$(C_{10}H_{16})H^+$	1	C10 bicycloalkanes
	$(C_{14}H_{24})H^+$	3	C14 bicycloalkanes
	$(C_{15}H_{26})H^+$	6	C15 bicycloalkanes
$(C_{n+6}H_{2n+6})H^+$	$(C_6H_6)H^+$	27	Benzene
	$(C_7H_8)H^+$	4	Toluene
	$(C_8H_{10})H^+$	0	C8 monoaromatics
	$(C_{14}H_{22})H^+$	4	C14 monoaromatics
	$(C_{15}H_{24})H^+$	6	C15 monoaromatics
$(C_nH_{2n+2}O)H^+$	$(CH_3OH)H^+$	9	Methanol
	$(C_2H_5OH)H^+$	37	Ethanol
$(C_nH_{2n}O)H^+$	$(CH_2O)H^+$	6	Formaldehyde
	$(C_2H_4O)H^+$	24	Acetaldehyde
	$(C_3H_6O)H^+$	21	Acetone/propanal
	$(C_4H_8O)H^+$	5	C4 carbonyl
$(C_{n+1}H_{2n}O)H^+$	$(C_3H_4O)H^+$	14	Acrolein
	$(C_4H_6O)H^+$	11	Methacrolein/crotonaldehyde
$(C_{n+3}H_{2n+2})H^+$	$(C_4H_4O)H^+$	1	Furan
$(C_{n+5}H_{2n+2})H^+$	$(C_6H_4O)H^+$	29	Epoxybenzene/Ethynylfuran/fragment
$(C_nH_{2n}O_2)H^+$	$(CH_2O_2)H^+$	53	Formic acid
	$(C_2H_4O_2)H^+$	0	Acetic acid
	$(C_3H_6O_2)H^+$	5	C3 acids
	$(C_4H_8O_2)H^+$	11	C4 acids

$C_{n+1}H_{2n}O_2)H^+$	$(C_2H_2O_2)H^+$	31	Glyoxal
	$(C_3H_4O_2)H^+$	7	Acrylic acid/ methylglyoxal/propanedial
	$(C_4H_6O_2)H^+$	2	C4 dial/dione/unsaturated acid
	$(C_5H_8O_2)H^+$	7	C5 dial/dione/unsaturated acid
$(C_{n+4}H_{2n+4}O_2)H^+$	$(C_5H_6O_2)H^+$	5	C5 esther/furan/unsaturated dialdehyde/lactone/dione derivative
$(C_{n+4}H_{2n+2}O_2)H^+$	$(C_5H_4O_2)H^+$	1	C5 furan/lactone/pyran derivative
$(C_{n+5}H_{2n+2}O_2)H^+$	$(C_6H_4O_2)H^+$	36	Benzoquinone
	$(C_5H_4O_3)H^+$	9	Furoic acid/Itaconic anhydride
	$(C_6H_6O_3)H^+$	5	Hydroxymethylfurfural/C6 anhydride
$(C_{n+3}H_{2n+2}O_4)H^+$	$(C_4H_4O_4)H^+$	1	Maleic acid
$(C_nH_{2n+1}NO_2)H^+$	$(CH_3NO_2)H^+$	75	Nitromethane
	$(C_2H_5NO_2)H^+$	33	Nitroethane
$(C_nH_{n+1}NO_2)H^+$	$(C_2H_3NO_2)H^+$	42	Nitroethylene
$(C_nH_{n+1}N)H^+$	$(C_2H_3N)H^+$	13	Acetonitrile

Table S4. Factor to species contribution ratios for the diesel cold urban cycle factor 3.

ion family	Ion formula	factor 3 contrib. to the signal (%)	Attribution
$C_nH_{2n}^+$	$C_2H_4^+$	2	ethene
$(C_nH_{2n})H^+$	$(C_3H_6)H^+$	2	Alkanes fragment
	$(C_4H_8)H^+$	32	Alkanes fragment
	$(C_5H_{10})H^+$	60	Alkanes fragment
	$(C_6H_{12})H^+$	70	Alkanes fragment
	$(C_7H_{14})H^+$	63	Alkanes fragment
	$(C_9H_{18})H^+$	76	C9 alkanes
	$(C_{10}H_{20})H^+$	74	C10 alkanes
	$(C_{11}H_{22})H^+$	59	C11 alkanes
$(C_{n+6}H_{2n+6})H^+$	$(C_4H_6)H^+$	30	Aldehydes fragment
	$(C_5H_8)H^+$	62	Aldehydes/cycloalkanes fragment
	$(C_6H_{10})H^+$	77	Aldehydes/cycloalkanes fragment
	$(C_7H_{12})H^+$	80	C7 cycloalkanes
	$(C_8H_{14})H^+$	67	C8 cycloalkanes
	$(C_9H_{16})H^+$	77	C9 cycloalkanes
	$(C_{10}H_{18})H^+$	83	C10 cycloalkanes
	$C_{11}H_{20})H^+$	76	C11 cycloalkanes
	$(C_{12}H_{22})H^+$	56	C12 cycloalkanes
	$(C_{13}H_{24})H^+$	38	C13 cycloalkanes
$(C_{n+5}H_{2n+7})H^+$	$(C_6H_8)H^+$	77	Bicycloalkanes fragment
	$(C_7H_{10})H^+$	71	Bicycloalkanes fragment
	$(C_8H_{12})H^+$	58	Bicycloalkanes fragment
	$(C_9H_{14})H^+$	64	C9 bicycloalkanes
	$(C_{10}H_{16})H^+$	81	C10 bicycloalkanes
	$(C_{11}H_{18})H^+$	75	C11 bicycloalkanes
	$(C_{12}H_{20})H^+$	62	C12 bicycloalkanes
	$(C_{13}H_{22})H^+$	46	C13 bicycloalkanes
	$(C_{14}H_{24})H^+$	27	C14 bicycloalkanes
	$(C_{15}H_{26})H^+$	14	C15 bicycloalkanes

$(C_{n+6}H_{2n+6})H^+$	$(C_7H_8)H^+$	57	Toluene
	$(C_8H_{10})H^+$	84	C8 monoaromatics
	$(C_9H_{12})H^+$	80	C9 monoaromatics
	$(C_{10}H_{14})H^+$	62	C10 monoaromatics
	$(C_{11}H_{16})H^+$	60	C11 monoaromatics
	$(C_{12}H_{18})H^+$	63	C12 monoaromatics
	$(C_{13}H_{20})H^+$	54	C13 monoaromatics
	$(C_{14}H_{22})H^+$	41	C14 monoaromatics
	$(C_{15}H_{24})H^+$	26	C15 monoaromatics
$(C_{n+9}H_{2n+10})H^+$	$(C_{10}H_{12})H^+$	68	C10 naphthenics monoaromatics
	$(C_{11}H_{14})H^+$	61	C11 naphthenics monoaromatics
	$(C_{12}H_{16})H^+$	49	C12 naphthenics monoaromatics
$(C_nH_{2n+2}O)H^+$	$(CH_3OH)H^+$	3	Methanol
	$(C_2H_5OH)H^+$	4	Ethanol
$(C_nH_{2n}O)H^+$	$(CH_2O)H^+$	1	Formaldehyde
	$(C_3H_6O)H^+$	65	Acetone/propanal
	$(C_4H_8O)H^+$	55	C4 carbonyls
	$(C_5H_{10}O)H^+$	51	C5 carbonyls
	$(C_6H_{12}O)H^+$	51	C6 carbonyls
	$(C_7H_{14}O)H^+$	56	C7 carbonyls
$(C_{n+1}H_{2n}O)H^+$	$(C_3H_4O)H^+$	1	Acrolein
	$(C_4H_6O)H^+$	45	Methacrolein/crotonaldehyde
	$(C_5H_8O)H^+$	74	C5 unsaturated carbonyls
	$(C_6H_{10}O)H^+$	70	C6 unsaturated carbonyls
$(C_{n+3}H_{2n+2})H^+$	$(C_4H_4O)H^+$	1	Furan
	$(C_5H_6O)H^+$	56	Methylfuran/pyran
	$(C_6H_8O)H^+$	55	dimethylfuran/ethylfuran/Cyclohexenone
$(C_{n+5}H_{2n+2})H^+$	$(C_6H_4O)H^+$	29	Epoxybenzene/ethynylfuran/fragment
	$(C_7H_6O)H^+$	47	Benzaldehyde/tropone
	$(C_8H_8O)H^+$	30	methylbenzaldehyde/phenylacetaldehyde/acetophenone
$(C_nH_{2n}O_2)H^+$	$(CH_2O_2)H^+$	15	Formic acid
	$(C_3H_6O_2)H^+$	16	C3 acids
	$(C_4H_8O_2)H^+$	10	C4 acids
$C_{n+1}H_{2n}O_2)H^+$	$(C_2H_2O_2)H^+$	38	Glyoxal
	$(C_3H_4O_2)H^+$	36	Acrylic acid/ methylglyoxal/propanedial
	$(C_4H_6O_2)H^+$	54	C4 dial/dione/unsaturated acid
	$(C_5H_8O_2)H^+$	34	C5 dial/dione/unsaturated acid
$(C_{n+4}H_{2n+4}O_2)H^+$	$(C_5H_6O_2)H^+$	24	C5 esther/furan/unsaturated dialdehyde/lactone/dione derivative
$(C_{n+4}H_{2n+2}O_2)H^+$	$(C_5H_4O_2)H^+$	39	C5 furan/lactone/pyran derivative
$(C_{n+5}H_{2n+2}O_2)H^+$	$(C_6H_4O_2)H^+$	27	Benzoquinone
	$(C_5H_4O_3)H^+$	10	Furoic acid/Itaconic anhydride
	$(C_6H_6O_3)H^+$	10	Hydroxymethylfurfural/C6 anhydride
$(C_{n+3}H_{2n+2}O_4)H^+$	$(C_4H_4O_4)H^+$	7	Maleic acid
$(C_nH_{2n+1}NO_2)H^+$	$(CH_3NO_2)H^+$	0	Nitromethane
	$(C_2H_5NO_2)H^+$	46	Nitroethane
$(C_nH_{n+1}NO_2)H^+$	$(C_2H_3NO_2)H^+$	35	Nitroethylene
$(C_nH_{n+1}N)H^+$	$(C_2H_3N)H^+$	54	Acetonitrile

Table S5. Factor to species contribution ratios for the diesel cold urban cycle factor 4.

ion family	Ion formula	factor 4 contrib. to the signal (%)	Attribution
$C_nH_{2n}^+$	$C_2H_4^+$		
$(C_nH_{2n})H^+$	$(C_3H_6)H^+$	2	Alkanes fragment
	$(C_4H_8)H^+$	10	Alkanes fragment
	$(C_5H_{10})H^+$	18	Alkanes fragment
	$(C_6H_{12})H^+$	24	Alkanes fragment
	$(C_7H_{14})H^+$	30	Alkanes fragment
	$(C_9H_{18})H^+$	22	C9 alkanes
	$(C_{10}H_{20})H^+$	26	C10 alkanes
	$(C_{11}H_{22})H^+$	41	C11 alkanes
$(C_{n+6}H_{2n+6})H^+$	$(C_3H_4)H^+$	3	Aldehydes fragment
	$(C_4H_6)H^+$	4	Aldehydes fragment
	$(C_5H_8)H^+$	16	Aldehydes/cycloalkanes fragment
	$(C_6H_{10})H^+$	16	Aldehydes/cycloalkanes fragment
	$(C_7H_{12})H^+$	13	C7 cycloalkanes
	$(C_8H_{14})H^+$	23	C8 cycloalkanes
	$(C_9H_{16})H^+$	16	C9 cycloalkanes
	$(C_{10}H_{18})H^+$	13	C10 cycloalkanes
	$C_{11}H_{20})H^+$	24	C11 cycloalkanes
	$(C_{12}H_{22})H^+$	44	C12 cycloalkanes
	$(C_{13}H_{24})H^+$	59	C13 cycloalkanes
$(C_{n+5}H_{2n+7})H^+$	$(C_6H_8)H^+$	15	Bicycloalkanes fragment
	$(C_7H_{10})H^+$	27	Bicycloalkanes fragment
	$(C_8H_{12})H^+$	41	Bicycloalkanes fragment
	$(C_9H_{14})H^+$	31	C9 bicycloalkanes
	$(C_{10}H_{16})H^+$	18	C10 bicycloalkanes
	$(C_{11}H_{18})H^+$	25	C11 bicycloalkanes
	$(C_{12}H_{20})H^+$	38	C12 bicycloalkanes
	$(C_{13}H_{22})H^+$	54	C13 bicycloalkanes
	$(C_{14}H_{24})H^+$	70	C14 bicycloalkanes
	$(C_{15}H_{26})H^+$	79	C15 bicycloalkanes
$(C_{n+6}H_{2n+6})H^+$	$(C_8H_{10})H^+$	0	C8 monoaromatics
	$(C_9H_{12})H^+$	14	C9 monoaromatics
	$(C_{10}H_{14})H^+$	34	C10 monoaromatics
	$(C_{11}H_{16})H^+$	38	C11 monoaromatics
	$(C_{12}H_{18})H^+$	35	C12 monoaromatics
	$(C_{13}H_{20})H^+$	43	C13 monoaromatics
	$(C_{14}H_{22})H^+$	53	C14 monoaromatics
	$(C_{15}H_{24})H^+$	66	C15 monoaromatics
$(C_{n+9}H_{2n+10})H^+$	$(C_{10}H_{12})H^+$	27	C10 naphthenics monoaromatics
	$(C_{11}H_{14})H^+$	38	C11 naphthenics monoaromatics
	$(C_{12}H_{16})H^+$	44	C12 naphthenics monoaromatics
$(C_nH_{2n+2}O)H^+$	$(CH_3OH)H^+$	4	Methanol
	$(C_2H_5OH)H^+$	6	Ethanol
$(C_nH_{2n}O)H^+$	$(CH_2O)H^+$	2	Formaldehyde

$(C_nH_{2n}O)H^+$	$(C_2H_4O)H^+$	8	Acetaldehyde
	$(C_3H_6O)H^+$	13	Acetone/propanal
	$(C_4H_8O)H^+$	39	C4 carbonyls
	$(C_5H_{10}O)H^+$	49	C5 carbonyls
	$(C_6H_{12}O)H^+$	49	C6 carbonyls
	$(C_7H_{14}O)H^+$	44	C7 carbonyls
$(C_{n+1}H_{2n}O)H^+$	$(C_3H_4O)H^+$	9	Acrolein
	$(C_4H_6O)H^+$	12	Methacrolein/crotonaldehyde
	$(C_5H_8O)H^+$	18	C5 unsaturated carbonyls
	$(C_6H_{10}O)H^+$	23	C6 unsaturated carbonyls
$(C_{n+3}H_{2n+2})H^+$	$(C_4H_4O)H^+$	2	Furan
	$(C_5H_6O)H^+$	28	Methylfurane/pyran
	$(C_6H_8O)H^+$	37	dimethylfuran/ethylfuran/Cyclohexenone
$(C_{n+5}H_{2n+2})H^+$	$(C_6H_4O)H^+$	37	Epoxybenzene/ethynylfuran/fragment
	$(C_7H_6O)H^+$	51	Benzaldehyde/tropone
	$(C_8H_8O)H^+$	65	methylbenzaldehyde/phenylacetaldehyde/acetophenone
$(C_nH_{2n}O_2)H^+$	$(CH_2O_2)H^+$	31	Formic acid
	$(C_2H_4O_2)H^+$	99	Acetic acid
	$(C_3H_6O_2)H^+$	73	C3 acids
	$(C_4H_8O_2)H^+$	73	C4 acids
$C_{n+1}H_{2n}O_2)H^+$	$(C_2H_2O_2)H^+$	24	Glyoxal
	$(C_3H_4O_2)H^+$	44	Acrylic acid/ methylglyoxal/propanedial
	$(C_4H_6O_2)H^+$	27	C4 dial/dione/unsaturated acid
	$(C_5H_8O_2)H^+$	47	C5 dial/dione/unsaturated acid
$(C_{n+4}H_{2n+4}O_2)H^+$	$(C_5H_6O_2)H^+$	69	C5 esther/furan/unsaturated dialdehyde/lactone/dione derivative
$(C_{n+4}H_{2n+2}O_2)H^+$	$(C_5H_4O_2)H^+$	60	C5 furan/lactone/pyran derivative
$(C_{n+5}H_{2n+2}O_2)H^+$	$(C_6H_4O_2)H^+$	37	Benzoquinone
$(C_{n+4}H_{2n+2}O_3)H^+$	$(C_4H_2O_3)H^+$	100	Maleic anhydride
	$(C_5H_4O_3)H^+$	81	Furoic acid/Itaconic anhydride
	$(C_6H_6O_3)H^+$	83	Hydroxymethylfurfural/C6 anhydride
$(C_{n+3}H_{2n+2}O_4)H^+$	$(C_4H_4O_4)H^+$	91	Maleic acid
$(C_nH_{2n+1}NO_2)H^+$	$(CH_3NO_2)H^+$	9	Nitromethane
	$(C_2H_5NO_2)H^+$	8	Nitroethane
$(C_nH_{n+1}NO_2)H^+$	$(C_2H_3NO_2)H^+$	6	Nitroethylene
$(C_nH_{n+1}N)H^+$	$(C_2H_3N)H^+$	2	Acetonitrile

Table S6. Factor to species contribution ratios for the diesel motorway cycle factor 1.

Ion family	Ion formula	Factor 1 contribution to the signal (%)	Attribution
$(C_nH_{2n}O_2)H^+$	$(C_2H_4O_2)H^+$	0	Acetic acid
(CnH2n+2O)H+	$(CH_3OH)H^+$	14	Methanol
$(C_nH_{2n})H^+$	$(C_3H_4)H^+$	31	Propene
	$(C_4H_6)H^+$	9	C4 alkenes
	$(C_5H_8)H^+$	5	C5 alkenes
	$(C_6H_{10})H^+$	2	C6 alkenes/Cyclohexane
$(C_{n+5}H_{2n+7})H^+$	$(C_{11}H_{18})H^+$	5	C11 bicycloalkane
	$(C_{12}H_{20})H^+$	1	C12 bicycloalkane
	$(C_{13}H_{22})H^+$	0	C13 bicycloalkane
$(C_nH_{2n}O)H^+$	$(CH_2O)H^+$	21	Formaldehyde
	$(C_2H_4O)H^+$	29	Acetaldehyde
	$(C_3H_6O)H^+$	15	Acetone/propanal
	$(C_4H_8O)H^+$	0	C4 carbonyls
	$(C_5H_{10}O)H^+$	0	C5 cabonyls
$(C_{n+2}H_{2n+2})H^+$	$(C_5H_8)H^+$	11	Cyclopentene/C5 diene
	$(C_6H_{10})H^+$	11	Cyclohexene/C6 diene
	$(C_7H_{12})H^+$	5	C7 cycloalkane
	$(C_8H_{14})H^+$	8	C8 cycloalkane
	$(C_9H_{16})H^+$	15	C9 cycloalkane
	$(C_{10}H_{18})H^+$	11	C10 cycloalkane
$(C_{n+6}H_{2n+6})H^+$	$(C_6H_6)H^+$	62	Benzene
	$(C_7H_8)H^+$	13	Toluene
	$(C_8H_{10})H^+$	19	Ethylbenzene/xylenes
	$(C_9H_{12})H^+$	12	C9 monoaromatics
	$(C_{10}H_{14})H^+$	8	C10 monoaromatics
$(C_nH_{2n+1}NO_2)H^+$	$(CH_3NO_2)H^+$	64	Nitromethane
$(C_{n+4}H_{2n+4}O_2)H^+$	$(C_5H_6O)H^+$	4	Cyclopentenone/furane/pyrane
$(C_{n+5}H_{2n+2})H^+$	$(C_6H_4O)H^+$	2	Epoxybenzene/ethynylfuran/fragment
$(C_{n+4}H_{2n+2}O_2)H^+$	$(C_5H_4O_2)H^+$	0	Furane/pyrone/lactone
$(C_{n+4}H_{2n+2}O_3)H^+$	$(C_4H_2O_3)H^+$	0	Maleic anhydride
$(C_{n+5}H_{2n+2}O_2)H^+$	$(C_6H_4O_2)H^+$	0	Benzoquinone
$(C_{n+7}H_{2n+2}O_3)H^+$	$(C_8H_4O_3)H^+$	0	Phthalic anhydride
$(C_{n+1}H_{2n}O)H^+$	$(C_3H_4O)H^+$	20	Acrolein
	$(C_4H_6O)H^+$	14	Methacrolein/crotonaldehyde

Table S7. Factor to species contribution ratios for the diesel motorway cycle factor 2.

Ion family	Ion formula	Factor 2 contribution to the signal (%)	Attribution
$(C_nH_{2n}O_2)H^+$	$(C_2H_4O_2)H^+$	2	Acetic acid
(CnH2n+2O)H+	$(CH_3OH)H^+$	24	Methanol
$(C_nH_{2n})H^+$	$(C_3H_4)H^+$	18	Alkane fragment
	$(C_4H_6)H^+$	21	Alkane fragment
	$(C_5H_8)H^+$	25	Alkane fragment
	$(C_6H_{10})H^+$	28	Alkane fragment
$(C_{n+5}H_{2n+7})H^+$	$(C_{11}H_{18})H^+$	36	C11 bicycloalkane
	$(C_{12}H_{20})H^+$	37	C12 bicycloalkane
	$(C_{13}H_{22})H^+$	30	C13 bicycloalkane
$(C_nH_{2n}O)H^+$	$(CH_2O)H^+$	8	Formaldehyde
	$(C_2H_4O)H^+$	16	Acetaldehyde
	$(C_3H_6O)H^+$	81	Acetone/propanal
	$(C_4H_8O)H^+$	91	C4 carbonyls
	$(C_5H_{10}O)H^+$	70	C5 carbonyls
$(C_{n+2}H_{2n+2})H^+$	$(C_5H_8)H^+$	19	Cycloalkane fragment
	$(C_6H_{10})H^+$	33	Cycloalkane fragment
	$(C_7H_{12})H^+$	32	C7 cycloalkane
	$(C_8H_{14})H^+$	28	C8 cycloalkane
	$(C_9H_{16})H^+$	30	C9 cycloalkane
	$(C_{10}H_{18})H^+$	37	C10 cycloalkane
$(C_{n+6}H_{2n+6})H^+$	$(C_6H_6)H^+$	0	Benzene
	$(C_7H_8)H^+$	4	Toluene
	$(C_8H_{10})H^+$	11	Ethylbenzene/xylenes
	$(C_9H_{12})H^+$	18	C9 monoaromatics
	$(C_{10}H_{14})H^+$	13	C10 monoaromatics
$(C_nH_{2n+1}NO_2)H^+$	$(CH_3NO_2)H^+$	13	Nitromethane
$(C_{n+4}H_{2n+4}O_2)H^+$	$(C_5H_6O)H^+$	0	Cyclopentenone/furane/pyrane
$(C_{n+5}H_{2n+2})H^+$	$(C_6H_4O)H^+$	4	Epoxybenzene/ethynylfuran/fragment
$(C_{n+4}H_{2n+2}O_2)H^+$	$(C_5H_4O_2)H^+$	0	Furane/pyrone/lactone
$(C_{n+4}H_{2n+2}O_3)H^+$	$(C_4H_2O_3)H^+$	0	Maleic anhydride
$(C_{n+5}H_{2n+2}O_2)H^+$	$(C_6H_4O_2)H^+$	2	Benzoquinone
$(C_{n+7}H_{2n+2}O_3)H^+$	$(C_8H_4O_3)H^+$	0	Phthalic anhydride
$(C_{n+1}H_{2n}O)H^+$	$(C_3H_4O)H^+$	3	Acrolein
	$(C_4H_6O)H^+$	15	Methacrolein/crotonaldehyde

Table S8. Factor to species contribution ratios for the diesel motorway cycle factor 3.

Ion family	Ion formula	Factor 3 contribution to the signal (%)	Attribution
$(C_nH_{2n}O_2)H^+$	$(C_2H_4O_2)H^+$	16	Acetic acid
(CnH2n+2O)H+	$(CH_3OH)H^+$	33	Methanol
$(C_nH_{2n})H^+$	$(C_3H_4)H^+$	38	Alkane fragment
	$(C_4H_6)H^+$	46	Alkane fragment
	$(C_5H_8)H^+$	47	Alkane fragment
	$(C_6H_{10})H^+$	50	Alkane fragment
$(C_{n+5}H_{2n+7})H^+$	$(C_{11}H_{18})H^+$	52	C11 bicycloalkane
	$(C_{12}H_{20})H^+$	59	C12 bicycloalkane
	$(C_{13}H_{22})H^+$	68	C13 bicycloalkane
$(C_nH_{2n}O)H^+$	$(CH_2O)H^+$	19	Formaldehyde
	$(C_2H_4O)H^+$	9	Acetaldehyde
	$(C_3H_6O)H^+$	1	Acetone/propanal
	$(C_4H_8O)H^+$	9	C4 carbonyls
	$(C_5H_{10}O)H^+$	24	C5 carbonyls
$(C_{n+2}H_{2n+2})H^+$	$(C_5H_8)H^+$	59	Cycloalkane fragment
	$(C_6H_{10})H^+$	56	Cycloalkane fragment
	$(C_7H_{12})H^+$	63	C7 cycloalkane
	$(C_8H_{14})H^+$	62	C8 cycloalkane
	$(C_9H_{16})H^+$	51	C9 cycloalkane
	$(C_{10}H_{18})H^+$	34	C10 cycloalkane
$(C_{n+6}H_{2n+6})H^+$	$(C_6H_6)H^+$	10	Benzene
	$(C_7H_8)H^+$	34	Toluene
	$(C_8H_{10})H^+$	48	Ethylbenzene/xylenes
	$(C_9H_{12})H^+$	60	C9 monoaromatics
	$(C_{10}H_{14})H^+$	77	C10 monoaromatics
$(C_nH_{2n+1}NO_2)H^+$	$(CH_3NO_2)H^+$	0	Nitromethane
$(C_{n+4}H_{2n+4}O_2)H^+$	$(C_5H_6O)H^+$	41	Cyclopentenone/furane/pyrane
$(C_{n+5}H_{2n+2})H^+$	$(C_6H_4O)H^+$	1	Epoxybenzene/ethynylfuran/fragment
$(C_{n+4}H_{2n+2}O_2)H^+$	$(C_5H_4O_2)H^+$	7	Furane/pyrone/lactone
$(C_{n+4}H_{2n+2}O_3)H^+$	$(C_4H_2O_3)H^+$	45	Maleic anhydride
$(C_{n+5}H_{2n+2}O_2)H^+$	$(C_6H_4O_2)H^+$	3	Benzoquinone
$(C_{n+7}H_{2n+2}O_3)H^+$	$(C_8H_4O_3)H^+$	8	Phthalic anhydride
$(C_{n+1}H_{2n}O)H^+$	$(C_3H_4O)H^+$	16	Acrolein
	$(C_4H_6O)H^+$	29	Methacrolein/crotonaldehyde

Table S9. Factor to species contribution ratios for the diesel motorway cycle factor 4.

Ion family	Ion formula	Factor 4 contribution to the signal (%)	Attribution
$(C_nH_{2n}O_2)H^+$	$(C_2H_4O_2)H^+$	14	Acetic acid
(CnH2n+2O)H+	$(CH_3OH)H^+$	23	Methanol
$(C_nH_{2n})H^+$	$(C_3H_4)H^+$	11	Alkane fragment
	$(C_4H_6)H^+$	23	Alkane fragment
	$(C_5H_8)H^+$	21	Alkane fragment
	$(C_6H_{10})H^+$	18	Alkane fragment
$(C_{n+5}H_{2n+7})H^+$	$(C_{11}H_{18})H^+$	3	C11 bicycloalkane
	$(C_{12}H_{20})H^+$	0	C12 bicycloalkane
	$(C_{13}H_{22})H^+$	0	C13 bicycloalkane
$(C_nH_{2n}O)H^+$	$(CH_2O)H^+$	12	Formaldehyde
	$(C_2H_4O)H^+$	46	Acetaldehyde
	$(C_3H_6O)H^+$	3	Acetone/propanal
	$(C_4H_8O)H^+$	0	C4 carbonyls
	$(C_5H_{10}O)H^+$	3	C5 carbonyls
$(C_{n+2}H_{2n+2})H^+$	$(C_5H_8)H^+$	10	Cyclopentene/C5 diene
	$(C_6H_{10})H^+$	0	Cyclohexene/C6 diene
	$(C_7H_{12})H^+$	0	C7 cycloalkane
	$(C_8H_{14})H^+$	0	C8 cycloalkane
	$(C_9H_{16})H^+$	0	C9 cycloalkane
	$(C_{10}H_{18})H^+$	14	C10 cycloalkane
$(C_{n+6}H_{2n+6})H^+$	$(C_6H_6)H^+$	27	Benzene
	$(C_7H_8)H^+$	35	Toluene
	$(C_8H_{10})H^+$	22	Ethylbenzene/xylenes
	$(C_9H_{12})H^+$	10	C9 monoaromatics
	$(C_{10}H_{14})H^+$	0	C10 monoaromatics
$(C_nH_{2n+1}NO_2)H^+$	$(CH_3NO_2)H^+$	0	Nitromethane
$(C_{n+4}H_{2n+4}O_2)H^+$	$(C_5H_6O)H^+$	49	Cyclopentenone/furane/pyrane
$(C_{n+5}H_{2n+2})H^+$	$(C_6H_4O)H^+$	32	Epoxybenzene/ethynylfuran/fragment
$(C_{n+4}H_{2n+2}O_2)H^+$	$(C_5H_4O_2)H^+$	69	Furane/pyrone/lactone
$(C_{n+4}H_{2n+2}O_3)H^+$	$(C_4H_2O_3)H^+$	55	Maleic anhydride
$(C_{n+5}H_{2n+2}O_2)H^+$	$(C_6H_4O_2)H^+$	75	Benzoquinone
$(C_{n+7}H_{2n+2}O_3)H^+$	$(C_8H_4O_3)H^+$	81	Phthalic anhydride
$(C_{n+1}H_{2n}O)H^+$	$(C_3H_4O)H^+$	59	Acrolein
	$(C_4H_6O)H^+$	42	Methacrolein/crotonaldehyde

Table S10. Factor to species contribution ratios for the diesel motorway cycle factor 5.

Ion family	Ion formula	Factor 5 contribution to the signal (%)	Attribution
$(C_nH_{2n}O_2)H^+$	$(C_2H_4O_2)H^+$	68	Acetic acid
(CnH2n+2O)H+	$(CH_3OH)H^+$	5	Methanol
$(C_nH_{2n})H^+$	$(C_3H_4)H^+$	3	Alkane fragment
	$(C_4H_6)H^+$	1	Alkane fragment
	$(C_5H_8)H^+$	1	Alkane fragment
	$(C_6H_{10})H^+$	2	Alkane fragment
$(C_{n+5}H_{2n+7})H^+$	$(C_{11}H_{18})H^+$	4	C11 bicycloalkane
	$(C_{12}H_{20})H^+$	3	C12 bicycloalkane
	$(C_{13}H_{22})H^+$	2	C13 bicycloalkane
$(C_nH_{2n}O)H^+$	$(CH_2O)H^+$	41	Formaldehyde
	$(C_2H_4O)H^+$	0	Acetaldehyde
	$(C_3H_6O)H^+$	0	Acetone/propanal
	$(C_4H_8O)H^+$	0	C4 carbonyls
	$(C_5H_{10}O)H^+$	3	C5 carbonyls
$(C_{n+2}H_{2n+2})H^+$	$(C_5H_8)H^+$	1	Cycloalkane fragment
	$(C_6H_{10})H^+$	0	Cycloalkane fragment
	$(C_7H_{12})H^+$	1	C7 cycloalkane
	$(C_8H_{14})H^+$	2	C8 cycloalkane
	$(C_9H_{16})H^+$	4	C9 cycloalkane
	$(C_{10}H_{18})H^+$	3	C10 cycloalkane
$(C_{n+6}H_{2n+6})H^+$	$(C_6H_6)H^+$	0	Benzene
	$(C_7H_8)H^+$	13	Toluene
	$(C_8H_{10})H^+$	0	Ethylbenzene/xylenes
	$(C_9H_{12})H^+$	0	C9 monoaromatics
	$(C_{10}H_{14})H^+$	2	C10 monoaromatics
$(C_nH_{2n+1}NO_2)H^+$	$(CH_3NO_2)H^+$	23	Nitromethane
$(C_{n+4}H_{2n+4}O_2)H^+$	$(C_5H_6O)H^+$	6	Cyclopentenone/furane/pyrane
$(C_{n+5}H_{2n+2})H^+$	$(C_6H_4O)H^+$	61	Epoxybenzene/ethynylfuran/fragment
$(C_{n+4}H_{2n+2}O_2)H^+$	$(C_5H_4O_2)H^+$	24	Furane/pyrone/lactone
$(C_{n+4}H_{2n+2}O_3)H^+$	$(C_4H_2O_3)H^+$	0	Maleic anhydride
$(C_{n+5}H_{2n+2}O_2)H^+$	$(C_6H_4O_2)H^+$	20	Benzoquinone
$(C_{n+7}H_{2n+2}O_3)H^+$	$(C_8H_4O_3)H^+$	11	Phthalic anhydride
$(C_{n+1}H_{2n}O)H^+$	$(C_3H_4O)H^+$	2	Acrolein
	$(C_4H_6O)H^+$	0	Methacrolein/crotonaldehyde

Table S11. Factor to species contribution ratios for the GDI cold urban cycle factor 1.

ion family	Ion formula	factor 1 contribution to the signal (%)	Attribution
$(C_nH_{2n+2}O)H^+$	$(CH_3OH)H^+$	11	Methanol
	$(C_2H_5OH)H^+$	0	Ethanol
$(C_{n+5}H_{2n+4}O)H^+$	$(C_6H_6O)H^+$	12	Phenol
$(C_nH_{2n}O)H^+$	$(CH_2O)H^+$	8	Formaldehyde
	$(C_2H_4O)H^+$	64	Acetaldehyde
	$(C_3H_6O)H^+$	26	Acetone
	$(C_4H_8O)H^+$	17	C4 carbonyls
	$(C_5H_{10}O)H^+$	24	C5 carbonyls
$C_nH_{2n}^+$	$C_2H_4^+$	75	Ethene/fragment
$(C_nH_{2n})H^+$	(C2H4)H+	15	Ethanol fragment
	$(C_3H_6)H^+$	76	Propene/fragment
	$(C_4H_8)H^+$	48	Butene/fragment
	$(C_5H_{10})H^+$	49	Pentene/fragment
	$(C_6H_{12})H^+$	46	Cyclohexane/Hexene/fragment
	$(C_7H_{14})H^+$	18	C7 Alkanes
	$(C_8H_{16})H^+$	10	C8 alkanes
	$(C_9H_{18})H^+$	4	C9 alkanes
$(C_{n+6}H_{2n+6})H^+$	$(C_6H_6)H^+$	13	Benzene
	$(C_7H_8)H^+$	16	Toluene
	$(C_8H_{10})H^+$	4	Ethylbenzene/xylene
	$(C_9H_{12})H^+$	0	C9 monoaromatics
	$(C_{10}H_{14})H^+$	0	C10 monoaromatics
	$(C_{11}H_{16})H^+$	0	C11 monoaromatics
$(C_{n+5}H_{2n+7})H^+$	$(C_4H_4)H^+$	69	1-buten-3-yne
	$(C_5H_6)H^+$	85	1-3-cyclopentadiene
	$(C_6H_8)H^+$	60	cycloxehadiene/methylcyclopentadiene
	$(C_7H_{10})H^+$	22	Fragment/cycloalkenes/Bicycloalkanes
	$(C_8H_{12})H^+$	5	Fragment/cycloalkenes/Bicycloalkanes
$(C_{n+2}H_{2n+2})H^+$	$(C_3H_4)H^+$	80	propadiene/fragment
	$(C_4H_6)H^+$	99	Butadiene/fragment
	$(C_5H_8)H^+$	75	Pentadiene/cyclopentane
	$(C_6H_{10})H^+$	61	Cyclohexane/fragment
	$(C_7H_{12})H^+$	33	C7 cycloalkanes
	$(C_8H_{14})H^+$	12	C8 cycloalkanes
	$(C_9H_{16})H^+$	4	C9 cycloalkanes
$(C_{n+7}H_{2n+4})H^+$	$(C_8H_6)H^+$	11	pentalene/fragment/phenylethyne
	$(C_9H_8)H^+$	1	Indene
	$(C_{10}H_{10})H^+$	0	Dihydronaphthalene
$(C_{n+9}H_{2n+6})H^+$	$(C_{10}H_8)H+$	0	Naphthalene
$(C_{n+9}H_{2n+10})H^+$	$(C_8H_8)H^+$	5	Styrene
	$(C_9H_{10})H^+$	3	Methylstyrene/Indane
	$(C_{10}H_{12})H^+$	0	Tetraline
$(C_{n+1}H_{2n+1}N)H^+$	$(C_2H_3N)H^+$	0	Acetonitrile
$(C_{n+2}H_{2n+1}N)H^+$	$(C_3H_3N)H^+$	6	Acrylonitrile
$(C_nH_{2n+1}NO_2)H^+$	$(CH_3NO_2)H^+$	14	Nitromethane
$(C_{n+1}H_{2n}O_4)H^+$	$(C_2H_2O_4)H^+$	5	Oxalic acid
$(C_{n+7}H_{2n+6}O)H^+$	$(C_8H_8O)H^+$	0	Acetophenone

$(C_{n+1}H_{2n}O)H^+$	$(C_3H_4O)H^+$	10	Acroleine
	$(C_4H_6O)H^+$	18	C4 unsaturated carbonyls
	$(C_5H_8O)H^+$	10	C5 unsaturated carbonyls
$(C_{n+6}H_{2n+4}O)H^+$	$(C_7H_6O)H^+$	0	benzaldehyde

Table S12. Factor to species contribution ratios for the GDI cold urban cycle factor 2.

ion family	Ion formula	factor 2 contribution to the signal (%)	Attribution
$(C_nH_{2n+2}O)H^+$	$(CH_3OH)H^+$	0	Methanol
	$(C_2H_5OH)H^+$	0	Ethanol
$(C_{n+5}H_{2n+4}O)H^+$	$(C_6H_6O)H^+$	15	Phenol
$(C_nH_{2n}O)H^+$	$(CH_2O)H^+$	0	Formaldehyde
	$(C_2H_4O)H^+$	0	Acetaldehyde
	$(C_3H_6O)H^+$	1	Acetone
	$(C_4H_8O)H^+$	0	C4 carbonyls
	$(C_5H_{10}O)H^+$	0	C5 carbonyls
$C_nH_{2n}^+$	$C_2H_4^+$	10	Ethene/fragment
$(C_nH_{2n})H^+$	(C2H4)H+	1	Ethene/fragment
	$(C_3H_6)H^+$	12	Propene/fragment
	$(C_4H_8)H^+$	26	Butene/fragment
	$(C_5H_{10})H^+$	25	Pentene/fragment
	$(C_6H_{12})H^+$	24	Cyclohexane/Hexene/fragment
	$(C_7H_{14})H^+$	30	C7 Alkanes
	$(C_8H_{16})H^+$	39	C8 alkanes
	$(C_9H_{18})H^+$	24	C9 alkanes
$(C_{n+6}H_{2n+6})H^+$	$(C_6H_6)H^+$	74	Benzene
	$(C_7H_8)H^+$	25	Toluene
	$(C_8H_{10})H^+$	8	Ethylbenzene/xylene
	$(C_9H_{12})H^+$	5	C9 monoaromatics
	$(C_{10}H_{14})H^+$	5	C10 monoaromatics
	$(C_{11}H_{16})H^+$	2	C11 monoaromatics
$(C_{n+5}H_{2n+7})H^+$	$(C_4H_4)H^+$	0	1-buten-3-yne
	$(C_5H_6)H^+$	0	1-3-cyclopentadiene
	$(C_6H_8)H^+$	3	cycloxehadiene/methylcyclopentadiene
	$(C_7H_{10})H^+$	10	Fragment/cycloalkenes/Bicycloalkanes
	$(C_8H_{12})H^+$	9	Fragment/cycloalkenes/Bicycloalkanes
$(C_{n+2}H_{2n+2})H^+$	$(C_3H_4)H^+$	4	propadiene/fragment
	$(C_4H_6)H^+$	0	Butadiene/fragment
	$(C_5H_8)H^+$	6	Pentadiene/cyclopentane
	$(C_6H_{10})H^+$	12	Cyclohexane/fragment
	$(C_7H_{12})H^+$	19	C7 cycloalkanes
	$(C_8H_{14})H^+$	33	C8 cycloalkanes
	$(C_9H_{16})H^+$	25	C9 cycloalkanes
$(C_{n+7}H_{2n+4})H^+$	$(C_8H_6)H^+$	0	pentalene/fragment/phenylethyne
	$(C_9H_8)H^+$	1	Indene
	$(C_{10}H_{10})H^+$	0	Dihydronaphthalene
$(C_{n+9}H_{2n+6})H^+$	$(C_{10}H_8)H+$	0	Naphthalene
$(C_{n+9}H_{2n+10})H^+$	$(C_8H_8)H^+$	2	Styrene
	$(C_9H_{10})H^+$	3	Methylstyrene/Indane
	$(C_{10}H_{12})H^+$	2	Tetraline
$(C_{n+1}H_{2n+1}N)H^+$	$(C_2H_3N)H^+$	3	Acetonitrile
$(C_{n+2}H_{2n+1}N)H^+$	$(C_3H_3N)H^+$	0	Acrylonitrile
$(C_nH_{2n+1}NO_2)H^+$	$(CH_3NO_2)H^+$	0	Nitromethane
$(C_{n+1}H_{2n}O_4)H^+$	$(C_2H_2O_4)H^+$	8	Oxalic acid
$(C_{n+7}H_{2n+6}O)H^+$	$(C_8H_8O)H^+$	5	Acetophenone

$(C_{n+1}H_{2n}O)H^+$	$(C_3H_4O)H^+$	0	Acroleine
	$(C_4H_6O)H^+$	0	C4 unsaturated carbonyls
	$(C_5H_8O)H^+$	6	C5 unsaturated carbonyls
$(C_{n+6}H_{2n+4}O)H^+$	$(C_7H_6O)H^+$	8	benzaldehyde

Table S13. Factor to species contribution ratios for the GDI cold urban cycle factor 3.

ion family	Ion formula	factor 3 contribution to the signal (%)	Attribution
$(C_nH_{2n+2}O)H^+$	$(CH_3OH)H^+$	76	Methanol
	$(C_2H_5OH)H^+$	90	Ethanol
$(C_{n+5}H_{2n+4}O)H^+$	$(C_6H_6O)H^+$	15	Phenol
$(C_nH_{2n}O)H^+$	$(CH_2O)H^+$	16	Formaldehyde
	$(C_2H_4O)H^+$	36	Acetaldehyde
	$(C_3H_6O)H^+$	68	Acetone
	$(C_4H_8O)H^+$	57	C4 carbonyls
	$(C_5H_{10}O)H^+$	49	C5 carbonyls
$C_nH_{2n}^+$	$C_2H_4^+$	0	Ethene/fragment
$(C_nH_{2n})H^+$	(C2H4)H+	64	Ethene/fragment
	$(C_3H_6)H^+$	5	Propene/fragment
	$(C_4H_8)H^+$	21	Butene/fragment
	$(C_5H_{10})H^+$	8	Pentene/fragment
	$(C_6H_{12})H^+$	12	Cyclohexane/Hexene/fragment
	$(C_7H_{14})H^+$	15	C7 Alkanes
	$(C_8H_{16})H^+$	20	C8 alkanes
	$(C_9H_{18})H^+$	3	C9 alkanes
$(C_{n+6}H_{2n+6})H^+$	$(C_6H_6)H^+$	7	Benzene
	$(C_7H_8)H^+$	48	Toluene
	$(C_8H_{10})H^+$	28	Ethylbenzene/xylene
	$(C_9H_{12})H^+$	3	C9 monoaromatics
	$(C_{10}H_{14})H^+$	4	C10 monoaromatics
	$(C_{11}H_{16})H^+$	7	C11 monoaromatics
$(C_{n+5}H_{2n+7})H^+$	$(C_4H_4)H^+$	5	1-buten-3-yne
	$(C_5H_6)H^+$	3	1-3-cyclopentadiene
	$(C_6H_8)H^+$	21	cycloxehadiene/methylcyclopentadiene
	$(C_7H_{10})H^+$	32	Fragment/cycloalkenes/Bicycloalkanes
	$(C_8H_{12})H^+$	10	Fragment/cycloalkenes/Bicycloalkanes
$(C_{n+2}H_{2n+2})H^+$	$(C_3H_4)H^+$	4	propadiene/fragment
	$(C_4H_6)H^+$	0	Butadiene/fragment
	$(C_5H_8)H^+$	10	Pentadiene/cyclopentane
	$(C_6H_{10})H^+$	19	Cyclohexane/fragment
	$(C_7H_{12})H^+$	34	C7 cycloalkanes
	$(C_8H_{14})H^+$	28	C8 cycloalkanes
	$(C_9H_{16})H^+$	12	C9 cycloalkanes
$(C_{n+7}H_{2n+4})H^+$	$(C_8H_6)H^+$	0	pentalene/fragment/phenylethyne
	$(C_9H_8)H^+$	10	Indene
	$(C_{10}H_{10})H^+$	10	Dihydronaphthalene
$(C_{n+9}H_{2n+6})H^+$	$(C_{10}H_8)H+$	6	Naphthalene
$(C_{n+9}H_{2n+10})H^+$	$(C_8H_8)H^+$	16	Styrene
	$(C_9H_{10})H^+$	3	Methylstyrene/Indane
	$(C_{10}H_{12})H^+$	8	Tetraline
$(C_{n+1}H_{2n+1}N)H^+$	$(C_2H_3N)H^+$	62	Acetonitrile
$(C_{n+2}H_{2n+1}N)H^+$	$(C_3H_3N)H^+$	27	Acrylonitrile
$(C_nH_{2n+1}NO_2)H^+$	$(CH_3NO_2)H^+$	36	Nitromethane
$(C_{n+1}H_{2n}O_4)H^+$	$(C_2H_2O_4)H^+$	26	Oxalic acid
$(C_{n+7}H_{2n+6}O)H^+$	$(C_8H_8O)H^+$	0	Acetophenone

$(C_{n+1}H_{2n}O)H^+$	$(C_3H_4O)H^+$	40	Acroleine
	$(C_4H_6O)H^+$	45	C4 unsaturated carbonyls
	$(C_5H_8O)H^+$	35	C5 unsaturated carbonyls
$(C_{n+6}H_{2n+4}O)H^+$	$(C_7H_6O)H^+$	19	benzaldehyde

Table S14. Factor to species contribution ratios for the GDI cold urban cycle factor 4.

ion family	Ion formula	factor 4 contribution to the signal (%)	Attribution
$(C_nH_{2n+2}O)H^+$	$(CH_3OH)H^+$	0	Methanol
	$(C_2H_5OH)H^+$	2	Ethanol
$(C_{n+5}H_{2n+4}O)H^+$	$(C_6H_6O)H^+$	3	Phenol
$(C_nH_{2n}O)H^+$	$(CH_2O)H^+$	3	Formaldehyde
	$(C_2H_4O)H^+$	0	Acetaldehyde
	$(C_3H_6O)H^+$	0	Acetone
	$(C_4H_8O)H^+$	0	C4 carbonyls
	$(C_5H_{10}O)H^+$	0	C5 carbonyls
$C_nH_{2n}^+$	$C_2H_4^+$	0	Ethene/fragment
$(C_nH_{2n})H^+$	(C2H4)H+	0	Ethene/fragment
	$(C_3H_6)H^+$	6	Propene/fragment
	$(C_4H_8)H^+$	4	Butene/fragment
	$(C_5H_{10})H^+$	14	Pentene/fragment
	$(C_6H_{12})H^+$	6	Cyclohexane/Hexene/fragment
	$(C_7H_{14})H^+$	0	C7 Alkanes
	$(C_8H_{16})H^+$	12	C8 alkanes
	$(C_9H_{18})H^+$	33	C9 alkanes
$(C_{n+6}H_{2n+6})H^+$	$(C_6H_6)H^+$	3	Benzene
	$(C_7H_8)H^+$	10	Toluene
	$(C_8H_{10})H^+$	51	Ethylbenzene/xylene
	$(C_9H_{12})H^+$	29	C9 monoaromatics
	$(C_{10}H_{14})H^+$	0	C10 monoaromatics
	$(C_{11}H_{16})H^+$	0	C11 monoaromatics
$(C_{n+5}H_{2n+7})H^+$	$(C_4H_4)H^+$	0	1-buten-3-yne
	$(C_5H_6)H^+$	0	1-3-cyclopentadiene
	$(C_6H_8)H^+$	0	cycloxehadiene/methylcyclopentadiene
	$(C_7H_{10})H^+$	3	Fragment/cycloalkenes/Bicycloalkanes
	$(C_8H_{12})H^+$	16	Fragment/cycloalkenes/Bicycloalkanes
$(C_{n+2}H_{2n+2})H^+$	$(C_3H_4)H^+$	3	propadiene/fragment
	$(C_4H_6)H^+$	0	Butadiene/fragment
	$(C_5H_8)H^+$	6	Pentadiene/cyclopentane
	$(C_6H_{10})H^+$	0	Cyclohexane/fragment
	$(C_7H_{12})H^+$	0	C7 cycloalkanes
	$(C_8H_{14})H^+$	14	C8 cycloalkanes
	$(C_9H_{16})H^+$	34	C9 cycloalkanes
$(C_{n+7}H_{2n+4})H^+$	$(C_8H_6)H^+$	19	pentalene/fragment/phenylethyne
	$(C_9H_8)H^+$	10	Indene
	$(C_{10}H_{10})H^+$	0	Dihydronaphthalene
$(C_{n+9}H_{2n+6})H^+$	$(C_{10}H_8)H+$	0	Naphthalene
$(C_{n+9}H_{2n+10})H^+$	$(C_8H_8)H^+$	36	Styrene
	$(C_9H_{10})H^+$	15	Methylstyrene/Indane
	$(C_{10}H_{12})H^+$	0	Tetraline
$(C_{n+1}H_{2n+1}N)H^+$	$(C_2H_3N)H^+$	17	Acetonitrile
$(C_{n+2}H_{2n+1}N)H^+$	$(C_3H_3N)H^+$	19	Acrylonitrile
$(C_nH_{2n+1}NO_2)H^+$	$(CH_3NO_2)H^+$	6	Nitromethane
$(C_{n+1}H_{2n}O_4)H^+$	$(C_2H_2O_4)H^+$	43	Oxalic acid
$(C_{n+7}H_{2n+6}O)H^+$	$(C_8H_8O)H^+$	19	Acetophenone

$(C_{n+1}H_{2n}O)H^+$	$(C_3H_4O)H^+$	15	Acroleine
	$(C_4H_6O)H^+$	9	C4 unsaturated carbonyls
	$(C_5H_8O)H^+$	12	C5 unsaturated carbonyls
$(C_{n+6}H_{2n+4}O)H^+$	$(C_7H_6O)H^+$	45	benzaldehyde

Table S15. Factor to species contribution ratios for the GDI cold urban cycle factor 5.

ion family	Ion formula	factor 5 contribution to the signal (%)	Attribution
$(C_nH_{2n+2}O)H^+$	$(CH_3OH)H^+$	1	Methanol
	$(C_2H_5OH)H^+$	8	Ethanol
$(C_{n+5}H_{2n+4}O)H^+$	$(C_6H_6O)H^+$	0	Phenol
$(C_nH_{2n}O)H^+$	$(CH_2O)H^+$	18	Formaldehyde
	$(C_2H_4O)H^+$	0	Acetaldehyde
	$(C_3H_6O)H^+$	4	Acetone
	$(C_4H_8O)H^+$	2	C4 carbonyls
	$(C_5H_{10}O)H^+$	0	C5 carbonyls
$C_nH_{2n}^+$	$C_2H_4^+$	3	Ethene/fragment
$(C_nH_{2n})H^+$	(C2H4)H+	0	Ethene/fragment
	$(C_3H_6)H^+$	2	Propene/fragment
	$(C_4H_8)H^+$	2	Butene/fragment
	$(C_5H_{10})H^+$	2	Pentene/fragment
	$(C_6H_{12})H^+$	9	Cyclohexane/Hexene/fragment
	$(C_7H_{14})H^+$	5	C7 Alkanes
	$(C_8H_{16})H^+$	0	C8 alkanes
	$(C_9H_{18})H^+$	8	C9 alkanes
$(C_{n+6}H_{2n+6})H^+$	$(C_6H_6)H^+$	3	Benzene
	$(C_7H_8)H^+$	0	Toluene
	$(C_8H_{10})H^+$	8	Ethylbenzene/xylene
	$(C_9H_{12})H^+$	56	C9 monoaromatics
	$(C_{10}H_{14})H^+$	57	C10 monoaromatics
	$(C_{11}H_{16})H^+$	19	C11 monoaromatics
$(C_{n+5}H_{2n+7})H^+$	$(C_4H_4)H^+$	0	1-buten-3-yne
	$(C_5H_6)H^+$	0	1-3-cyclopentadiene
	$(C_6H_8)H^+$	1	cycloxehadiene/methylcyclopentadiene
	$(C_7H_{10})H^+$	1	Fragment/cycloalkenes/Bicycloalkanes
	$(C_8H_{12})H^+$	7	Fragment/cycloalkenes/Bicycloalkanes
$(C_{n+2}H_{2n+2})H^+$	$(C_3H_4)H^+$	0	propadiene/fragment
	$(C_4H_6)H^+$	0	Butadiene/fragment
	$(C_5H_8)H^+$	0	Pentadiene/cyclopentane
	$(C_6H_{10})H^+$	3	Cyclohexane/fragment
	$(C_7H_{12})H^+$	2	C7 cycloalkanes
	$(C_8H_{14})H^+$	0	C8 cycloalkanes
	$(C_9H_{16})H^+$	3	C9 cycloalkanes
$(C_{n+7}H_{2n+4})H^+$	$(C_8H_6)H^+$	14	pentalene/fragment/phenylethyne
	$(C_9H_8)H^+$	58	Indene
	$(C_{10}H_{10})H^+$	25	Dihydronaphthalene
$(C_{n+9}H_{2n+6})H^+$	$(C_{10}H_8)H+$	11	Naphthalene
$(C_{n+9}H_{2n+10})H^+$	$(C_8H_8)H^+$	32	Styrene
	$(C_9H_{10})H^+$	45	Methylstyrene/Indane
	$(C_{10}H_{12})H^+$	40	Tetraline
$(C_{n+1}H_{2n+1}N)H^+$	$(C_2H_3N)H^+$	18	Acetonitrile
$(C_{n+2}H_{2n+1}N)H^+$	$(C_3H_3N)H^+$	20	Acrylonitrile
$(C_nH_{2n+1}NO_2)H^+$	$(CH_3NO_2)H^+$	5	Nitromethane
$(C_{n+1}H_{2n}O_4)H^+$	$(C_2H_2O_4)H^+$	5	Oxalic acid
$(C_{n+7}H_{2n+6}O)H^+$	$(C_8H_8O)H^+$	47	Acetophenone

$(C_{n+1}H_{2n}O)H^+$	$(C_3H_4O)H^+$	18	Acroleine
	$(C_4H_6O)H^+$	12	C4 unsaturated carbonyls
	$(C_5H_8O)H^+$	7	C5 unsaturated carbonyls
$(C_{n+6}H_{2n+4}O)H^+$	$(C_7H_6O)H^+$	10	benzaldehyde

Table S16. Factor to species contribution ratios for the GDI cold urban cycle factor 6.

ion family	Ion formula	factor 6 contribution to the signal (%)	Attribution
$(C_nH_{2n+2}O)H^+$	$(CH_3OH)H^+$	12	Methanol
	$(C_2H_5OH)H^+$	0	Ethanol
$(C_{n+5}H_{2n+4}O)H^+$	$(C_6H_6O)H^+$	55	Phenol
$(C_nH_{2n}O)H^+$	$(CH_2O)H^+$	55	Formaldehyde
	$(C_2H_4O)H^+$	0	Acetaldehyde
	$(C_3H_6O)H^+$	2	Acetone
	$(C_4H_8O)H^+$	24	C4 carbonyls
	$(C_5H_{10}O)H^+$	27	C5 carbonyls
$C_nH_{2n}^+$	$C_2H_4^+$	13	Ethene/fragment
$(C_nH_{2n})H^+$	(C2H4)H+	20	Ethene/fragment
	$(C_3H_6)H^+$	0	Propene/fragment
	$(C_4H_8)H^+$	0	Butene/fragment
	$(C_5H_{10})H^+$	2	Pentene/fragment
	$(C_6H_{12})H^+$	4	Cyclohexane/Hexene/fragment
	$(C_7H_{14})H^+$	31	C7 Alkanes
	$(C_8H_{16})H^+$	19	C8 alkanes
	$(C_9H_{18})H^+$	29	C9 alkanes
$(C_{n+6}H_{2n+6})H^+$	$(C_6H_6)H^+$	0	Benzene
	$(C_7H_8)H^+$	0	Toluene
	$(C_8H_{10})H^+$	0	Ethylbenzene/xylene
	$(C_9H_{12})H^+$	6	C9 monoaromatics
	$(C_{10}H_{14})H^+$	34	C10 monoaromatics
	$(C_{11}H_{16})H^+$	72	C11 monoaromatics
$(C_{n+5}H_{2n+7})H^+$	$(C_4H_4)H^+$	26	1-buten-3-yne
	$(C_5H_6)H^+$	12	1-3-cyclopentadiene
	$(C_6H_8)H^+$	15	cycloxehadiene/methylcyclopentadiene
	$(C_7H_{10})H^+$	33	Fragment/cycloalkenes/Bicycloalkanes
	$(C_8H_{12})H^+$	53	Fragment/cycloalkenes/Bicycloalkanes
$(C_{n+2}H_{2n+2})H^+$	$(C_3H_4)H^+$	10	propadiene/fragment
	$(C_4H_6)H^+$	1	Butadiene/fragment
	$(C_5H_8)H^+$	3	Pentadiene/cyclopentane
	$(C_6H_{10})H^+$	5	Cyclohexane/fragment
	$(C_7H_{12})H^+$	12	C7 cycloalkanes
	$(C_8H_{14})H^+$	13	C8 cycloalkanes
	$(C_9H_{16})H^+$	22	C9 cycloalkanes
$(C_{n+7}H_{2n+4})H^+$	$(C_8H_6)H^+$	55	pentalene/fragment/phenylethyne
	$(C_9H_8)H^+$	20	Indene
	$(C_{10}H_{10})H^+$	65	Dihydronaphthalene
$(C_{n+9}H_{2n+6})H^+$	$(C_{10}H_8)H+$	84	Naphthalene
$(C_{n+9}H_{2n+10})H^+$	$(C_8H_8)H^+$	9	Styrene
	$(C_9H_{10})H^+$	32	Methylstyrene/Indane
	$(C_{10}H_{12})H^+$	50	Tetraline
$(C_{n+1}H_{2n+1}N)H^+$	$(C_2H_3N)H^+$	1	Acetonitrile
$(C_{n+2}H_{2n+1}N)H^+$	$(C_3H_3N)H^+$	27	Acrylonitrile
$(C_nH_{2n+1}NO_2)H^+$	$(CH_3NO_2)H^+$	39	Nitromethane
$(C_{n+1}H_{2n}O_4)H^+$	$(C_2H_2O_4)H^+$	13	Oxalic acid
$(C_{n+7}H_{2n+6}O)H^+$	$(C_8H_8O)H^+$	28	Acetophenone

$(C_{n+1}H_{2n}O)H^+$	$(C_3H_4O)H^+$	17	Acroleine
	$(C_4H_6O)H^+$	16	C4 unsaturated carbonyls
	$(C_5H_8O)H^+$	31	C5 unsaturated carbonyls
$(C_{n+6}H_{2n+4}O)H^+$	$(C_7H_6O)H^+$	17	benzaldehyde

Table S17. Detailed emission factors for individual NMVOCs for the GDI vehicle cold urban cycle.

	GDI cold urban NMVOC EFs			
Chemical family	Ion formula	EFs (µg/km)	ΔEF (µg/km)	Attribution
Alcohols	$(CH_3OH)H^+$	287	71	Methanol
	$(C_2H_5OH)H^+$	2110	1066	Ethanol
Bicycloalkanes	$(C_7H_{10})H^+$	106	34	Fragment/cycloalkenes/Bicycloalkanes
	$(C_8H_{12})H^+$	102	38	Fragment/cycloalkenes/Bicycloalkanes
Branched alkanes	C_5H_{12}	225	50	C5 ramifié
	C_6H_{14}	312	53	C6 ramifié
	C_6H_{14}	1009	161	C6 ramifié
	C_7H_{16}	243	44	C7 ramifié
	C_7H_{16}	1302	233	C7 ramifié
	C_8H_{18}	1186	492	C8 ramifié
	C_8H_{18}	357	96	C8 ramifié
	C_8H_{18}	2720	671	C8 ramifié
	C_8H_{18}	1008	626	C8 ramifié
	C_8H_{18}	105	30	C8 ramifié
	C_8H_{18}	480	120	C8 ramifié
	C_8H_{18}	496	77	C8 ramifié
	C_8H_{18}	3282	587	C8 ramifié
	C_8H_{18}	2397	409	C8 ramifié
	C_9H_{20}	2178	466	C9 ramifié
	C_9H_{20}	182	34	C9 ramifié
	C_9H_{20}	396	73	C9 ramifié
	C_9H_{20}	846	171	C9 ramifié
	C_9H_{20}	2506	503	C9 ramifié
	C_9H_{20}	139	29	C9 ramifié
	C_9H_{20}	147	14	C9 ramifié
	C_9H_{20}	258	57	C9 ramifié
	C_9H_{20}	371	86	C9 ramifié
	C_9H_{20}	6950	1538	C9 ramifié
	C_9H_{20}	16253	3822	C9 ramifié
	$C_{10}H_{22}$	456	113	C10 ramifié
	$C_{10}H_{22}$	1152	299	C10 ramifié
	$C_{10}H_{22}$	606	186	C10 ramifié
	$C_{11}H_{24}$	129	40	C11 ramifié
	$C_{10}H_{22}$	174	57	C10 ramifié
	$C_{11}H_{24}$	608	227	C11 ramifié
	$C_{11}H_{24}$	703	249	C11 ramifié
	$C_{10}H_{22}$	1212	482	C10 ramifié
	$C_{10}H_{22}$	213	101	C10 ramifié
	$C_{10}H_{22}$	142	59	C10 ramifié
	$C_{10}H_{22}$	282	127	C10 ramifié
	$C_{11}H_{24}$	550	300	C11 ramifié
	$C_{11}H_{24}$	371	176	C11 ramifié
	$C_{10}H_{22}$	223	120	C10 ramifié
	$C_{10}H_{22}$	395	276	C11 ramifié
	$C_{12}H_{26}$	2723	1904	C12 ramifié
	$C_{12}H_{26}$	335	248	C12 ramifié

Branched alkanes	$C_{12}H_{26}$	821	543	C12 ramifié
	$C_{13}H_{28}$	144	112	C13 ramifié
	$C_{12}H_{26}$	27	22	C12 ramifié
	$C_{12}H_{26}$	843	626	C12 ramifié
	$C_{13}H_{28}$	413	322	C13 ramifié
	$C_{13}H_{28}$	33	29	C13 ramifié
	$C_{13}H_{28}$	38	32	C13 ramifié
	$C_{13}H_{28}$	277	226	C13 ramifié
	$C_{12}H_{26}$	36	32	C12 ramifié
	$C_{12}H_{26}$	24	21	C13 ramifié
	$C_{13}H_{28}$	32	33	C13 ramifié
	$C_{13}H_{28}$	29	32	C13 ramifié
Carbonyls	$(CH_2O)H^+$	304	71	Formaldehyde
	$(C_2H_4O)H^+$	1485	591	Acetaldehyde
	$(C_3H_6O)H^+$	580	196	Acetone
	$(C_4H_8O)H^+$	101	37	
	$(C_5H_{10}O)H^+$	84	40	
Cycloalkanes	$(C_6H_{12})H^+$	1514	717	Cyclohexane
	C_7H_{14}	40	8	cyclopentane, 1,3-dimethyl-
	C_7H_{14}	81	19	cyclopentane, 1,2-dimethyl-, cis-
	C_7H_{14}	32	6	cyclohexane, methyl-
	C_7H_{14}	189	27	cyclohexane, methyl-
	C_8H_{16}	17	10	C3 cyclopentane
	C_8H_{16}	151	36	C3 cyclopentane
	C_8H_{16}	255	52	C3 cyclopentane
	C_8H_{16}	21	13	cyclohexane, 1,2-dimethyl-, cis-
	C_8H_{16}	515	119	cyclohexane, ethyl-
	C_9H_{18}	61	15	cyclohexane, a,b,c-trimethyl-
	C_9H_{18}	174	27	cyclopentane, 1-methyl-2-propyl-
	C_9H_{18}	46	12	cyclohexane, propyl-
	C_9H_{18}	32	15	cyclopentane, butyl-
Dihydronaphthalenes	$(C_9H_8)H^+$	337	146	Indene
	$(C_{10}H_{10})H^+$	100	46	Dihydronaphthalene
Linear alkanes	C_5H_{12}	142	51	pentane
	C_6H_{14}	1894	397	hexane
	C_7H_{16}	767	163	heptane
	C_8H_{18}	5209	952	octane
	C_9H_{20}	3460	883	nonane
	$C_{10}H_{22}$	162	89	decane
	$C_{11}H_{24}$	46	39	undecane
	$C_{12}H_{26}$	7	5	dodecane
	$C_{13}H_{28}$	0	0	tridecane
	$C_{14}H_{30}$	6	4	tetradecane
	$C_{15}H_{32}$	5	4	pentadecane
	$C_{16}H_{34}$	4	4	hexadecane
	$C_{17}H_{36}$	5	2	heptadecane
	$C_{18}H_{38}$	6	1	octadecane
	$C_{19}H_{40}$	3	2	nonadecane
	$C_{20}H_{42}$	3	2	eicosane

Linear alkanes	$C_{21}H_{44}$	6	2	heneicosane
	$C_{22}H_{46}$	11	1	docosane
Monoaromatics	C_6H_6	12878	1744	benzene
	C_7H_8	15155	1730	Toluene
	C_8H_{10}	9018	1603	ethylbenzene
	C_8H_{10}	29336	4237	m+p-xylene
	C_8H_{10}	12964	2275	o-xylene
	C_9H_{12}	23661	6160	benzene, propyl
	C_9H_{12}	10945	2563	benzene, 1-ethyl-3-methyl-
	C_9H_{12}	4250	879	benzene, 1-ethyl-4-methyl-
	C_9H_{12}	4583	1144	benzene, 1,3,5-trimethyl-
	C_9H_{12}	3788	976	benzene, 1-ethyl-2-methyl-
	C_9H_{12}	1226	730	benzene, 1-ethenyl-2/3/4-methyl-
	C_9H_{12}	13245	4226	benzene, 1,2,4-trimethyl-
	C_9H_{12}	2466	772	benzene, 1,2,3-trimethyl-
Monoaromatics	$C_{10}H_{14}$	438	150	benzene, 1-methyl-3-propyl-
	$C_{10}H_{14}$	711	272	benzene, butyl-
	$C_{10}H_{14}$	1400	518	benzene, 1-ethyl-3,5/1,4/1,3/1,2/2,4/2,4-dimethyl-
	$C_{10}H_{14}$	715	354	benzene, 1-ethyl-3,5-dimethyl-
	$C_{10}H_{14}$	713	330	benzene, 1-ethyl-2,4-dimethyl-
	$C_{10}H_{14}$	1094	509	benzene, 2-ethyl-1,3-dimethyl-
	$C_{10}H_{12}$	85	58	benzene, 4-ethenyl-1,2-dimethyl-
	$C_{10}H_{14}$	270	140	benzene,1-ethyl-2,3-dimethyl-
	$C_{10}H_{14}$	411	268	benzene, 1,2,4,5-tetramethyl-
	$C_{10}H_{14}$	640	402	benzene, 1,2,3,5-tetramethyl-
	$C_{10}H_{14}$	126	105	benzene, 1,2,3,4-tetramethyl-
	$(C_{11}H_{16})H^+$	171	69	C11 monoaromatics
Naphthalenes	$C_{10}H_8$	949	735	naphthalene
Naphthenics monoaromatics	$(C_8H_8)H^+$	1605	491	Styrene
	$(C_9H_{10})H^+$	1661	542	Methylstyrene/Indane
	$(C_{10}H_{12})H^+$	399	143	Tetraline
Nitrogen compounds	$(C_2H_3N)H^+$	201	117	Acetonitrile
	$(C_3H_3N)H^+$	44	19	Acrylonitrile
	$(CH_3NO_2)H^+$	74	17	Nitromethane
Other oxygenated	$(C_6H_6O)H^+$	44	15	Phenol
	$(C_2H_2O_4)H^+$	187	75	Oxalic acid
	$(C_8H_8O)H^+$	897	321	Acetophenone
	$(C_7H_6O)H^+$	1064	416	benzaldehyde
Unsaturated aliphatics	$(C_4H_4)H^+$	150	29	1-buten-3-yne
	$(C_5H_6)H^+$	315	80	1-3-cyclopentadiene
	$(C_6H_8)H^+$	252	75	cycloxehadiene/methylcyclopentadiene
	$C_2H_4^+$	158	50	Ethene
	$(C_3H_6)H^+$	3903	1326	Propene/fragment
	$(C_4H_8)H^+$	6398	2860	Butene/fragment
	$(C_5H_{10})H^+$	2808	1245	Pentene/fragment
	$(C_4H_6)H^+$	842	266	Butadiene
	$(C_5H_8)H^+$	744	263	Cyclopentene
	$(C_6H_{10})H^+$	445	202	Cyclohexene/cyclohexane fragment
	$(C_8H_6)H^+$	56	20	pentalene/fragment/phenylethyne

	$(C_3H_4O)H^+$	283	53	Acroleine
Unsaturated carbonyls	$(C_4H_6O)H^+$	260	68	C4 unsaturated carbonyls
	$(C_5H_8O)H^+$	116	63	C5 unsaturated carbonyls

Table S18. Detailed emission factors for individual NMVOCs for the GDI vehicle motorway cycle.

GDI motorway NMVOC EFs				
Chemical family	Ion formula	EFs (µg/km)	ΔEF (µg/km)	Attribution
Branched alkanes	C_5H_{12}	84	25	C5 ramifié
	C_6H_{14}	69	22	C6 ramifié
	C_6H_{14}	223	75	C6 ramifié
	C_7H_{16}	143	62	C7 ramifié
	C_7H_{16}	139	71	C7 ramifié
	C_8H_{18}	936	93	C8 ramifié
	C_8H_{18}	83	32	C8 ramifié
	C_8H_{18}	221	105	C8 ramifié
	C_8H_{18}	203	81	C8 ramifié
	C_8H_{18}	23	9	C8 ramifié
	C_8H_{18}	90	40	C8 ramifié
	C_8H_{18}	45	23	C8 ramifié
	C_8H_{18}	171	92	C8 ramifié
	C_8H_{18}	140	77	C8 ramifié
	C_9H_{20}	232	105	C9 ramifié
	C_9H_{20}	7	10	C9 ramifié
	C_9H_{20}	25	14	C9 ramifié
	C_9H_{20}	48	28	C9 ramifié
	C_9H_{20}	138	77	C9 ramifié
	C_9H_{20}	6	8	C9 ramifié
	C_9H_{20}	12	7	C9 ramifié
	C_9H_{20}	8	11	C9 ramifié
	C_9H_{20}	18	10	C9 ramifié
	C_9H_{20}	351	199	C9 ramifié
	C_9H_{20}	799	453	C9 ramifié
	$C_{10}H_{22}$	28	14	C10 ramifié
	$C_{10}H_{22}$	67	29	C10 ramifié
	$C_{10}H_{22}$	34	28	C10 ramifié
	$C_{11}H_{24}$	6	8	C11 ramifié
	$C_{11}H_{24}$	10	9	C10 ramifié
	$C_{11}H_{24}$	39	30	C11 ramifié
	$C_{11}H_{24}$	46	37	C11 ramifié
	$C_{10}H_{22}$	118	64	C10 ramifié
	$C_{10}H_{22}$	20	16	C10 ramifié
	$C_{11}H_{24}$	24	19	C10 ramifié
	$C_{11}H_{24}$	59	42	C11 ramifié
	$C_{11}H_{24}$	33	26	C11 ramifié
	$C_{10}H_{22}$	13	19	C10 ramifié
	$C_{10}H_{22}$	49	42	C10 ramifié
	$C_{12}H_{26}$	379	219	C12 ramifié
	$C_{12}H_{26}$	52	32	C12 ramifié
	$C_{12}H_{26}$	106	61	C12 ramifié
	$C_{13}H_{28}$	20	16	C12 ramifié
	$C_{12}H_{26}$	3	4	C12 ramifié
	$C_{12}H_{26}$	129	70	C12 ramifié
	$C_{13}H_{28}$	86	50	C13 ramifié

Branched alkanes	$C_{13}H_{28}$	4	6	C13 ramifié
	$C_{13}H_{28}$	4	5	C13 ramifié
	$C_{13}H_{28}$	46	30	C13 ramifié
	$C_{12}H_{26}$	4	5	C13 ramifié
	$C_{13}H_{28}$	11	6	C13 ramifié
	$C_{13}H_{28}$	11	6	C13 ramifié
Carbonyls	$(C_2H_4O)H^+$	142	97	Acetaldehyde,
Cycloalkanes	C_7H_{14}	9	12	cyclopentane, 1,2-dimethyl-, cis-
	C_7H_{14}	55	59	cyclohexane, methyl-
	C_8H_{16}	7	10	cyclopentane, 1-ethyl-3-methyl-, trans-
	C_8H_{16}	17	25	cyclohexane, ethyl-
Dihydronaphthalenes	$(C_8H_8)H^+$	130	62	styrene, phenetyl acetate
Linear alkanes	C_5H_{12}	50	12	pentane
	C_6H_{14}	216	84	hexane
	C_7H_{16}	47	25	heptane
	C_8H_{18}	247	136	octane
	C_9H_{20}	212	116	nonane
	$C_{10}H_{22}$	21	13	decane
	$C_{11}H_{24}$	5	7	undecane
	$C_{12}H_{26}$	2	2	dodecane
	$C_{13}H_{28}$	1	2	tridecane
	$C_{14}H_{30}$	4	3	tetradecane
	$C_{15}H_{32}$	3	2	pentadecane
	$C_{16}H_{34}$	3	2	hexadecane
	$C_{17}H_{36}$	3	2	heptadecane
	$C_{18}H_{38}$	3	2	octadecane
	$C_{19}H_{40}$	0	0	nonadecane
	$C_{20}H_{42}$	1	2	eicosane
	$C_{21}H_{44}$	4	3	heneicosane
	$C_{22}H_{46}$	7	2	docosane
Monoaromatics	C_6H_6	1655	517	benzene
	C_7H_8	890	461	Toluene
	C_8H_{10}	272	192	ethylbenzene
	C_8H_{10}	1291	805	m+p-xylene
	C_8H_{10}	481	309	o-xylene
	C_9H_{12}	767	613	benzene, propyl
	C_9H_{12}	394	273	benzene, 1-ethyl-3-methyl-
	C_9H_{12}	174	120	benzene, 1-ethyl-4-methyl-
	C_9H_{12}	203	135	benzene, 1,3,5-trimethyl-
	C_9H_{12}	141	99	benzene, 1-ethyl-2-methyl-
	C_9H_{10}	115	57	benzene, 1-ethenyl-2/3/4-methyl-
	C_9H_{12}	804	504	benzene, 1,2,4-trimethyl-
	C_9H_{12}	147	97	benzene, 1,2,3-trimethyl-
	$C_{10}H_{14}$	27	38	benzene, 1-methyl-3-propyl-
	$C_{10}H_{14}$	39	56	benzene, butyl-
	$C_{10}H_{14}$	75	65	benzene, 1-ethyl-3,5/1,4/1,3/1,2/2,4/2,4-dimethyl-
	$C_{10}H_{14}$	44	36	benzene, 1-ethyl-3,5-dimethyl-
	$C_{10}H_{14}$	45	39	benzene, 1-ethyl-2,4-dimethyl-

Monoaromatics	$C_{10}H_{14}$	72	61	benzene, 2-ethyl-1,3-dimethyl-
	$C_{10}H_{14}$	16	23	benzene,1-ethyl-2,3-dimethyl-
	$C_{10}H_{14}$	45	41	benzene, 1,2,4,5-tetramethyl-
	$C_{10}H_{14}$	70	63	benzene, 1,2,3,5-tetramethyl-
	$C_{10}H_{14}$	12	17	benzene, 1,2,3,4-tetramethyl-
Naphthalenes	$C_{10}H_8$	277	118	naphthalene
Naphthenics monoaromatics	$(C_{10}H_{12})H^+$	49	23	1,2,3,4-tetrahydronaphthalene, ethyl styrene, methyl propenylbenzene
Nitrogen compounds	$(C_2H_3N)H^+$	62	26	acetonitrile
Other oxygenated	$(C_7H_6O)H^+$	70	40	benzaldehyde
	$(C_8H_8O)H^+$	82	44	Acetophenone

Table S19. Detailed emission factors for individual NMVOCs for the PFI vehicle cold urban cycle.

PFI cold urban NMVOC EFs			
Chemical family	Ion formula	Efs (µg/km)	Attribution
Acids	$(C_2H_4O_2)H^+$	3	Acetic acid
	$(C_3H_6O_2)H^+$	2	propanoic acid
	$(C_4H_8O_2)H^+$	1	butanoic acid
	$(C_5H_{10}O_2)H^+$	2	pentanoic acid
Alcohols	$(CH_3OH)H^+$	58	Methanol
	$(C_2H_5OH)H^+$	292	Ethanol
Carbonyls	$(CH_2O)H^+$	35	Formaldehyde
	$(C_2H_4O)H^+$	1062	Acetaldehyde
	$(C_3H_6O)H^+$	179	Acetone
	$(C_4H_8O)H^+$	33	Butanone
	$(C_5H_{10}O)H^+$	28	Pentanone
	$(C_6H_{12}O)H^+$	12	Hexanone
	$(C_7H_{14}O)H^+$	3	Heptanone
	$(C_8H_{16}O)H^+$	8	Octanone
Cycloalkanes	C6H12	333	Cyclohexane
	C_7H_{14}	32	Methylcyclohexane
	C_8H_{16}	17	Ethylcyclohexane
	C_9H_{18}	7	Propylcyclohexane
	$C_{10}H_{20}$	2	Butylcyclohexane
	$C_{11}H_{22}$	3	Pentylcyclohexane
	$C_{12}H_{24}$	3	Hexylcyclohexane
	$C_{13}H_{26}$	3	Hepthylcyclohexane
	$C_{14}H_{28}$	4	Octylcyclohexane
	$C_{15}H_{30}$	3	Nonylcyclohexane
Dihydronaphthalenes	$(C_{10}H_{10})H^+$	9	Dihydronaphthalene
	$(C_{11}H_{12})H^+$	4	ethylindene/dimethylindene
	$(C_9H_8)H^+$	22	Indene
Linear alkanes	C_6H_{14}	1279	Hexane
	C_7H_{16}	597	Heptane
	C_8H_{18}	579	Octane
	C_9H_{20}	485	Nonane
	$C_{10}H_{22}$	156	Decane
	$C_{11}H_{24}$	120	Undecane
	$C_{12}H_{26}$	103	Dodecane
	$C_{13}H_{28}$	26	Tridecane
	$C_{14}H_{30}$	89	Tetradecane
	$C_{15}H_{32}$	69	Pentadecane
	$C_{16}H_{34}$	42	Hexadecane
	$C_{17}H_{36}$	23	Heptadecane
	$C_{18}H_{38}$	12	Octadecane
	$C_{19}H_{40}$	13	Nonadecane
	$C_{20}H_{42}$	6	Eicosane
	$C_{21}H_{44}$	8	Heneicosane
	$C_{22}H_{46}$	11	Docosane
Monoaromatics	C_6H_6	1708	Benzène
	C_7H_8	3593	Toluene
	C_8H_{10}	3454	(m+p)Xylene

Monoaromatics	C_8H_{10}	1422	o-Xylene
	C_8H_{10}	1307	Ethylbenzene
	C_9H_{12}	1250	benzene, 1,2,4-trimethyl-
	C_9H_{12}	885	benzene, 1-ethyl-4-methyl-
	C_9H_{12}	435	benzene, 1-ethyl-2-methyl-
	C_9H_{12}	434	benzene, 1,3,5-trimethyl-
	C_9H_{12}	343	benzene, 1-ethenyl-2/3/4-methyl-
	C_9H_{12}	274	benzene, 1,2,3-trimethyl-
	C_9H_{12}	59	benzene, propyl
	C_9H_{12}	15	benzene, 1-ethyl-3-methyl-
	$C_{10}H_{14}$	115	benzene, 2-ethyl-1,3-dimethyl-
	$C_{10}H_{14}$	110	benzene, 1-ethyl-3,5/1,4/1,3/1,2/2,4/2,4-dimethyl-
	$C_{10}H_{14}$	95	benzene, 1,2,3,5-tetramethyl-
	$C_{10}H_{14}$	89	benzene, 1-ethyl-2,4-dimethyl-
	$C_{10}H_{14}$	60	benzene, 1,2,4,5-tetramethyl-
	$C_{10}H_{14}$	59	benzene, 1-ethyl-3,5-dimethyl-
	$C_{10}H_{14}$	32	benzene, 1,2,3,4-tetramethyl-
	$C_{10}H_{14}$	21	benzene,1-ethyl-2,3-dimethyl-
	$C_{10}H_{14}$	19	benzene, butyl-
	$C_{10}H_{14}$	12	Benzene, a ethyl b,c dimethyl
	$C_{10}H_{14}$	10	Benzene, a methyl b (c methylethyl)
	$C_{10}H_{14}$	7	Benzene, a methyl b propyl
	$(C_{11}H_{16})H^+$	31	C11 monoaromatics
	$(C_{12}H_{18})H^+$	9	C12 monoaromatics
	$(C_{13}H_{20})H^+$	4	C13 monoaromatics
	$(C_{14}H_{22})H^+$	2	C15 monoaromatics
	$(C_{15}H_{24})H^+$	2	C14 monoaromatics
	$(C_{16}H_{26})H^+$	1	C16 monoaromatics
Naphthalenes	$C_{10}H_8$	291	Naphtalene
	$C_{11}H_{10}$	15	1-methylnaphthalene
	$C_{12}H_{12}$	3	dimethylnaphthalene/ethylnaphthalene
Naphthenics monoaromatics	$(C_8H_8)H^+$	121	Styrene
	$(C_9H_{10})H^+$	104	Indane
	$(C_{10}H_{12})H^+$	38	Tetrahydronaphthalene, ethyl styrene
	$(C_{11}H_{14})H^+$	18	methyltetralin
	$(C_{12}H_{16})H^+$	8	dimethyltetraline/ethyltetraline
Nitrogen compounds	$(C_2H_3N)H^+$	31	Acetonitrile
	$(C_3H_3N)H^+$	6	Acrylonitrile
	$(CH_3NO_2)H^+$	15	Nitromethane
Other oxygenated	$(C_2H_2O_4)H^+$	17	oxalic acid
	$(C_7H_6O_2)H^+$	1	Acide benzoïque
	$(C_6H_6O)H^+$	4	Phenol
	$(C_7H_8O)H^+$	2	methylphenol
	$(C_3H_4O_2)H^+$	1	Acide acrylique/methylglyoxal
	$(C_4H_6O_2)H^+$	2	crotonic acid/ butanedione
	$(C_6H_8O_2)H^+$	1	sorbic acid/cyclohexanedione
	$(C_8H_{10}O)H^+$	2	Ethylphenol
	$(C_5H_6O)H^+$	6	Methyl furan
	$(C_6H_8O)H^+$	2	Ethylfuran/dimethylfuran/cyclohexenone
	$(C_6H_6O_2)H^+$	1	Benzene diol/acetylfuran
Other oxygenated	$(C_7H_6O)H^+$	363	Benzaldehyde

	$(C_8H_8O)H^+$	107	Acetophenone
Unsaturated aliphatics	$(C_4H_4)H^+$	10	1-buten-3-yne
	$(C_5H_6)H^+$	89	cyclopentadiene
	$(C_5H_8)H^+$	399	C5 dienes/Fragment
	$(C_6H_8)H^+$	61	cyclohexadiène/methylcyclopentadiène
	$(C_7H_{10})H^+$	21	
Unsaturated carbonyls	$(C_3H_4O)H^+$	54	Acrolein
	$(C_4H_6O)H^+$	56	methacrolein
	$(C_5H_8O)H^+$	28	C5 unsaturated carbonyls
	$(C_6H_{10}O)H^+$	12	C6 unsaturated carbonyls
	$(C_7H_{12}O)H^+$	2	C7 unsaturated aldehydes

Table S20. Detailed emission factors for individual NMVOCs for the PFII vehicle motorway cycle.

PFI motorway NMVOC EFs				
Chemical family	Ion formula	EFs (µg/km)	ΔEF (µg/km)	Attribution
Alcohols	$(CH_3OH)H^+$	1	1	Methanol
Carbonyls	$(C_2H_4O)H^+$	6	5	Acetaldehyde
	$(C_3H_6O)H^+$	4	3	Acetone/propanal
	$(C_4H_8O)H^+$	1	1	C4 carbonyls
Dihydronaphthalenes	$(C_9H_8)H^+$	2	0	Indene
Linear alkanes	C_6H_{14}	14		Hexane
	C_7H_{16}	7		Heptane
	C_8H_{18}	8		Octane
	C_9H_{20}	8		Nonane
	$C_{10}H_{22}$	1		Decane
	$C_{11}H_{24}$	2		Undecane
	$C_{12}H_{26}$	2		Dodecane
	$C_{13}H_{28}$	2		Tridecane
	$C_{14}H_{30}$	7		Tetradecane
	$C_{15}H_{32}$	6		Pentadecane
	$C_{16}H_{34}$	3		Hexadecane
	$C_{17}H_{36}$	3		Heptadecane
	$C_{18}H_{38}$	3		Octadecane
	$C_{19}H_{40}$	4		Nonadecane
	$C_{20}H_{42}$	1		Eicosane
	$C_{21}H_{44}$	2		Heneicosane
	$C_{22}H_{46}$	3		Docosane
Monoaromatics	$(C_{11}H_{16})H^+$	1	0	C11 monoaromatics
	C_6H_6	37		Benzène
	C_7H_8	64		Toluene
	C_8H_{10}	20		Ethylbenzene
	C_8H_{10}	69		(m+p)Xylene
	C_8H_{10}	23		o-Xylene
	C_9H_{12}	17		benzene, 1-ethyl-4-methyl-
	C_9H_{12}	12		benzene, 1,3,5-trimethyl-
	C_9H_{12}	11		benzene, 1-ethyl-2-methyl-
	C_9H_{12}	7		benzene, 1-ethenyl-2/3/4-methyl-
	C_9H_{12}	26		benzene, 1,2,4-trimethyl-
	C_9H_{12}	4		benzene, 1,2,3-trimethyl-
	$C_{10}H_{14}$	13	5	C10 monoaromatics
Naphthalenes	$C_{10}H_8$	1	1	Naphthalene
Naphthenics monoaromatics	$(C_8H_8)H^+$	9	2	styrene, phenetyl acetate
	$(C_9H_{10})H^+$	9	3	indane, methylstyrene
	$(C_{10}H_{12})H^+$	4	1	Tetraline
Other oxygenated	$(C_7H_6O)H^+$	4	2	benzaldehyde
	$(C_8H_8O)H^+$	8	3	Acetophenone
Unsaturated carbonyls	$(C_3H_4O)H^+$	3	1	Acrolein
	$(C_4H_6O)H^+$	3	1	C4 unsaturated carbonyls

Table S21. Detailed emission factors for individual NMVOCs for the diesel vehicle cold urban cycle.

Diesel cold urban cycle NMVOC EFs				
Chemical family	Ion formula	EFs (µg/km)	ΔEF (µg/km)	Attribution
Acids	$(CH_2O_2)H^+$	37	9	Formic acid
	$(C_2H_4O_2)H^+$	2710	2810	Acetic acid
	$(C_3H_6O_2)H^+$	50	39	C3 acids
	$(C_4H_8O_2)H^+$	7	5	C4 acids
Alcohols	$(CH_3OH)H^+$	494	112	Methanol
	$(C_2H_5OH)H^+$	11	4	Ethanol
Alkanes	$(C_6H_{12})H^+$	32	24	C6 alkanes
	$(C_7H_{14})H^+$	33	24	C7 alkanes
	$(C_8H_{16})H^+$	53	38	C8 alkanes
	$(C_9H_{18})H^+$	204	145	C9 alkanes
	$(C_{10}H_{20})H^+$	372	63	C10 alkanes
	$(C_{11}H_{22})H^+$	229	63	C11 alkanes
	$(C_{12}H_{24})H^+$	138	54	C12 alkanes
	$(C_{13}H_{26})H^+$	215	138	C13 alkanes
	$(C_{14}H_{28})H^+$	236	126	C14 alkanes
	$(C_{15}H_{30})H^+$	121	78	C15 alkanes
	$(C_{16}H_{32})H^+$	83	48	C16 alkanes
Bicycloalkanes	$(C_8H_{12})H^+$	6	3	C8 bicycloalkanes
	$(C_9H_{14})H^+$	9	4	C9 bicycloalkanes
	$(C_{10}H_{16})H^+$	19	7	C10 bicycloalkanes
	$(C_{11}H_{18})H^+$	21	8	C11 bicycloalkanes
	$(C_{12}H_{20})H^+$	18	7	C12 bicycloalkanes
	$(C_{13}H_{22})H^+$	13	7	C13 bicycloalkanes
	$(C_{14}H_{24})H^+$	7	5	C14 bicycloalkanes
	$(C_{15}H_{26})H^+$	4	3	C15 bicycloalkanes
Carbonyls	$(CH_2O)H^+$	723	73	Formaldehyde
	$(C_2H_4O)H^+$	3191	726	Acetaldehyde
	$(C_3H_6O)H^+$	2051	627	Acetone/propanal
	$(C_4H_8O)H^+$	174	94	C4 carbonyls
	$(C_5H_{10}O)H^+$	39	24	C5 carbonyls
	$(C_6H_{12}O)H^+$	10	6	C6 carbonyls
	$(C_7H_{14}O)H^+$	4	2	C7 carbonyls
Cycloalkanes	$(C_7H_{12})H^+$	81	26	C7 cycloalkanes
	$(C_8H_{14})H^+$	76	26	C8 cycloalkanes
	$(C_9H_{16})H^+$	67	20	C9 cycloalkanes
	$(C_{10}H_{18})H^+$	31	9	C10 cycloalkanes
	$C_{11}H_{20})H^+$	12	4	C11 cycloalkanes
	$(C_{12}H_{22})H^+$	5	2	C12 cycloalkanes
	$(C_{13}H_{24})H^+$	3	2	C13 cycloalkanes
Monoaromatics	$(C_6H_6)H^+$	380	90	Benzene
	$(C_7H_8)H^+$	33	13	Toluene
	$(C_8H_{10})H^+$	51	23	C8 monoaromatics
	$(C_9H_{12})H^+$	55	25	C9 monoaromatics
	$(C_{10}H_{14})H^+$	29	15	C10 monoaromatics
	$(C_{11}H_{16})H^+$	12	6	C11 monoaromatics
	$(C_{12}H_{18})H^+$	7	3	C12 monoaromatics

Monoaromatics	$(C_{13}H_{20})H^+$	5	3	C13 monoaromatics
	$(C_{14}H_{22})H^+$	4	2	C14 monoaromatics
	$(C_{15}H_{24})H^+$	3	2	C15 monoaromatics
Naphthenics monoaromatics	$(C_{10}H_{12})H^+$	5	3	C10 naphthenics monoaromatics
	$(C_{11}H_{14})H^+$	5	3	C11 naphthenics monoaromatics
	$(C_{12}H_{16})H^+$	2	1	C12 naphthenics monoaromatics
Nitrogen compounds	$(CH_3NO_2)H^+$	893	223	Nitromethane
	$(C_2H_5NO_2)H^+$	16	5	Nitroethane
	$(C_2H_3NO_2)H^+$	4	1	Nitroethylene
	$(C_2H_3N)H^+$	28	11	Acetonitrile
Other oxygenated	$(C_4H_4O)H^+$	24	5	Furan
	$(C_5H_6O)H^+$	6	3	Methylfurane/pyran
	$(C_6H_8O)H^+$	4	2	dimethylfuran/ethylfuran/Cyclohexenone
	$(C_6H_4O)H^+$	8	4	Epoxybenzene/ethynylfuran/fragment
	$(C_7H_6O)H^+$	6	5	Benzaldehyde/tropone
	$(C_8H_8O)H^+$	4	4	methylbenzaldehyde/phenylacetaldehyde/acetophenone
	$(C_2H_2O_2)H^+$	10	11	Glyoxal
	$(C_3H_4O_2)H^+$	16	11	Acrylic acid/ methylglyoxal/propanedial
	$(C_4H_6O_2)H^+$	48	27	C4 dial/dione/unsaturated acid
	$(C_5H_8O_2)H^+$	5	3	C5 dial/dione/unsaturated acid
	$(C_5H_6O_2)H^+$	6	4	C5 esther/furan/unsaturated dialdehyde/lactone/dione derivative
	$(C_5H_4O_2)H^+$	8	5	C5 furan/lactone/pyran derivative
	$(C_6H_4O_2)H^+$	4	1	Benzoquinone
	$(C_4H_2O_3)H^+$	58	58	Maleic anhydride
	$(C_5H_4O_3)H^+$	12	9	Furoic acid/Itaconic anhydride
	$(C_6H_6O_3)H^+$	5	4	Hydroxymethylfurfural/C6 anhydride
	$(C_4H_4O_4)H^+$	3	2	Maleic acid
Unsaturated aliphatics	$C_2H_4^+$	22	25	Ethene
	$(C_3H_6)H^+$	1041	277	Propene
	$(C_4H_8)H^+$	274	80	C4 alkenes
	$(C_5H_{10})H^+$	37	13	C5 alkenes
	$(C_6H_{12})H^+$	5	2	C6 alkenes/Cyclohexane
	$(C_4H_6)H^+$	12	10	Butadiene/C4 aldehydes fragment
	$(C_5H_8)H^+$	16	5	C5 diene/cyclopentene/C5 aldehydes fragment
	$(C_6H_{10})H^+$	8	3	C6 diene/cyclohexene/C6 aldehydes fragment
Unsaturated carbonyls	$(C_3H_4O)H^+$	431	172	Acrolein
	$(C_4H_6O)H^+$	96	44	Methacrolein/crotonaldehyde
	$(C_5H_8O)H^+$	15	7	C5 unsaturated carbonyls
	$(C_6H_{10}O)H^+$	5	2	C6 unsaturated carbonyls

Table S22. Detailed emission factors for individual NMVOCs for the diesel vehicle motorway cycle.

Diesel motorway cycle NMVOC EFs				
Chemical family	Ion formula	EFs (µg/km)	ΔEF (µg/km)	Attribution
Acids	$(C_2H_4O_2)H^+$	456	257	Acetic acid
Alcohols	$(CH_3OH)H^+$	31	15	Methanol
Bicycloalkanes	$(C_{11}H_{18})H^+$	4	0	C11 bicycloalkane
	$(C_{12}H_{20})H^+$	4	0	C12 bicycloalkane
	$(C_{13}H_{22})H^+$	3	0	C13 bicycloalkane
Carbonyls	$(CH_2O)H^+$	9	0	Formaldehyde
	$(C_2H_4O)H^+$	341	0	Acetaldehyde
	$(C_3H_6O)H^+$	364	0	Acetone/propanal
	$(C_4H_8O)H^+$	34	0	C4 carbonyls
	$(C_5H_{10}O)H^+$	11	0	C5 cabonyls
Cycloalkanes	$(C_7H_{12})H^+$	2	0	C7 cycloalkane
	$(C_8H_{14})H^+$	2	0	C8 cycloalkane
	$(C_9H_{16})H^+$	2	0	C9 cycloalkane
	$(C_{10}H_{18})H^+$	2	0	C10 cycloalkane
Linear alkanes	C_7H_{16}	10	7	Heptane
	C_8H_{18}	15	11	Octane
	C_9H_{20}	54	38	Nonane
	$C_{10}H_{22}$	93	16	Decane
	$C_{11}H_{24}$	54	15	Undecane
	$C_{12}H_{26}$	31	12	Dodecane
	$C_{13}H_{28}$	46	30	Tridecane
	$C_{14}H_{30}$	49	26	Tetradecane
	$C_{15}H_{32}$	24	15	Pentadecane
	$C_{16}H_{34}$	16	9	Hexadecane
	$C_{17}H_{36}$	18	10	Heptadecane
	$C_{18}H_{38}$	16	12	Octadecane
	$C_{19}H_{40}$	6	4	Nonadecane
	$C_{20}H_{42}$	8	6	Eicosane
	$C_{21}H_{44}$	12	8	Heneicosane
	$C_{22}H_{46}$	18	7	Docosane
Monoaromatics	$(C_6H_6)H^+$	28	0	Benzene
	$(C_7H_8)H^+$	5	0	Toluene
	$(C_8H_{10})H^+$	6	0	Ethylbenzene/xylenes
	$(C_9H_{12})H^+$	10	0	C9 monoaromatics
	$(C_{10}H_{14})H^+$	7	0	C10 monoaromatics
Nitrogen compounds	$(CH_3NO_2)H^+$	96	0	Nitromethane
Other oxygenated	$(C_5H_6O)H^+$	4	0	Cyclopentenone/furane/pyrane
	$(C_6H_4O)H^+$	2	0	Epoxybenzene/ethynylfuran/fragment
	$(C_5H_4O_2)H^+$	10	0	Furane/pyrone/lactone
	$(C_4H_2O_3)H^+$	79	0	Maleic anhydride
	$(C_6H_4O_2)H^+$	8	0	Benzoquinone
	$(C_8H_4O_3)H^+$	7	0	Phthalic anhydride
Unsaturated aliphatics	$(C_3H_6)H^+$	13	7	Propene
	$(C_4H_8)H^+$	16	7	C4 alkenes
	$(C_5H_{10})H^+$	10	5	C5 alkenes
	$(C_6H_{12})H^+$	7	3	C6 alkenes/Cyclohexane

Unsaturated aliphatics	$(C_5H_8)H^+$	4	0	Cyclopentene/C5 diene
	$(C_6H_{10})H^+$	3	0	Cyclohexene/C6 diene
Unsaturated carbonyls	$(C_3H_4O)H^+$	50	0	Acrolein
	$(C_4H_6O)H^+$	20	0	Methacrolein/crotonaldehyde

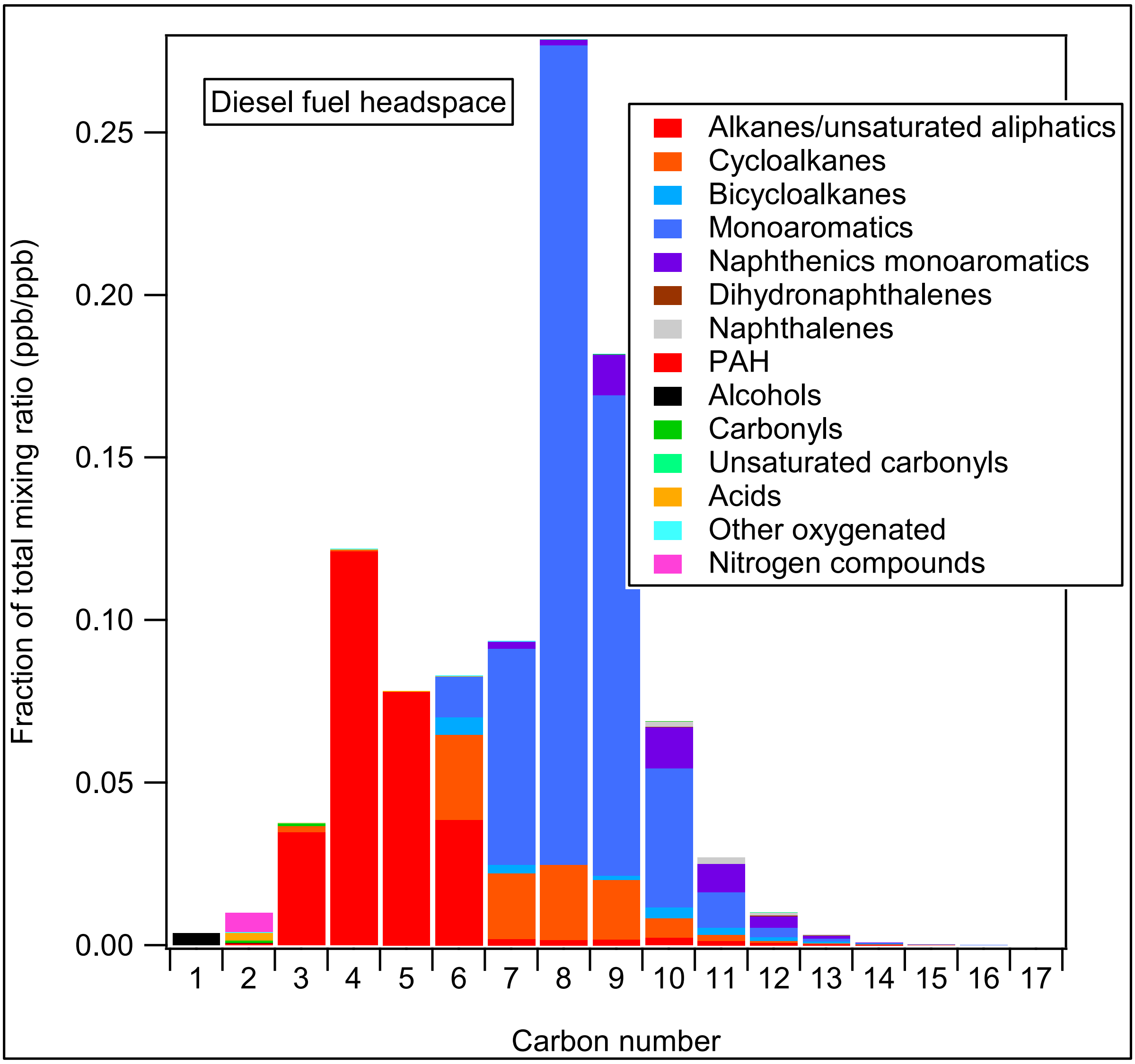

Figure S1. Fraction of total mixing ratio (ppb/ppb) by mean of carbon number for the diesel fuel headspace. Colors correspond to the main chemical classes measured by PTR-ToF-MS.

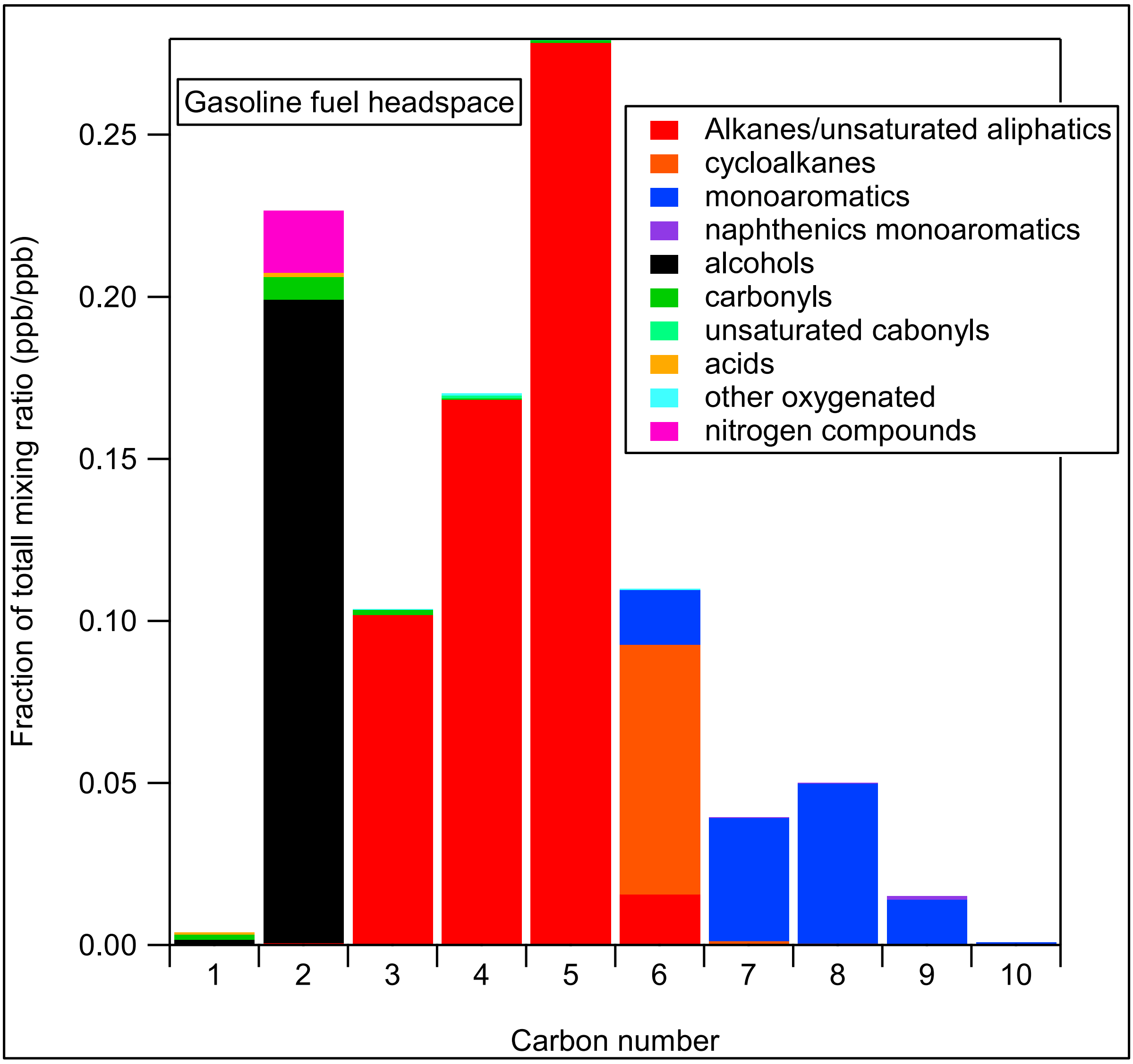

Figure S2. Fraction of total mixing ratio (ppb/ppb) by mean of carbon number for the gasoline SP95-E10 fuel headspace. Colors correspond to the main chemical classes measured by PTR-ToF-MS.

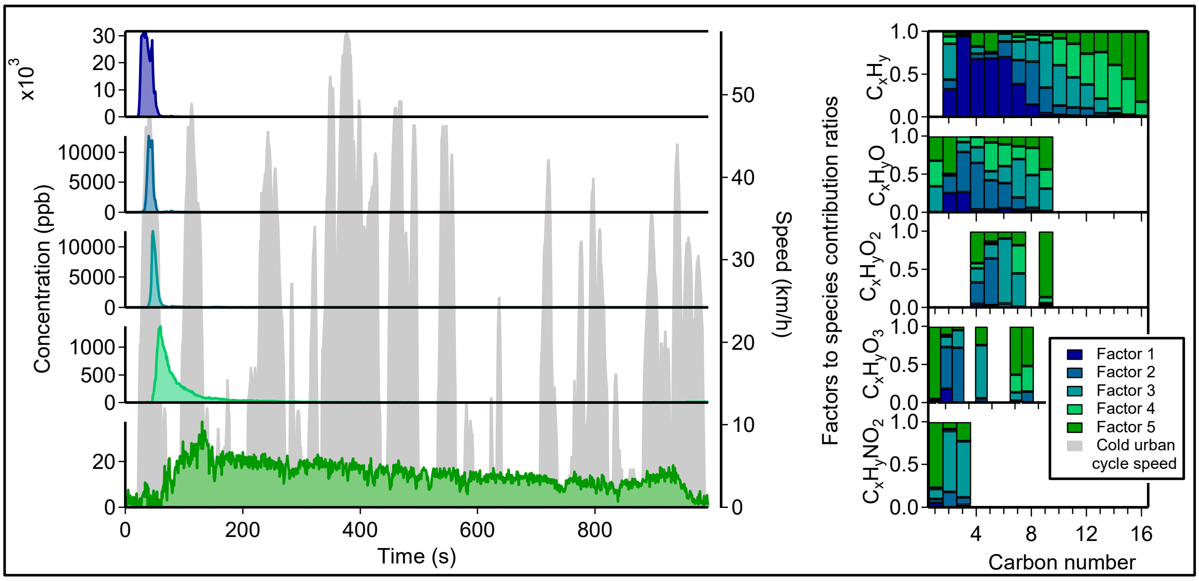

Figure S3. PMF factors temporal variations and contribution ratios for a typical PFI vehicle cold urban cycle. The grey zone represents speed variations during the cold urban cycle. Factors to species contribution ratios are classified by mean of carbon, oxygen, and nitrogen number.

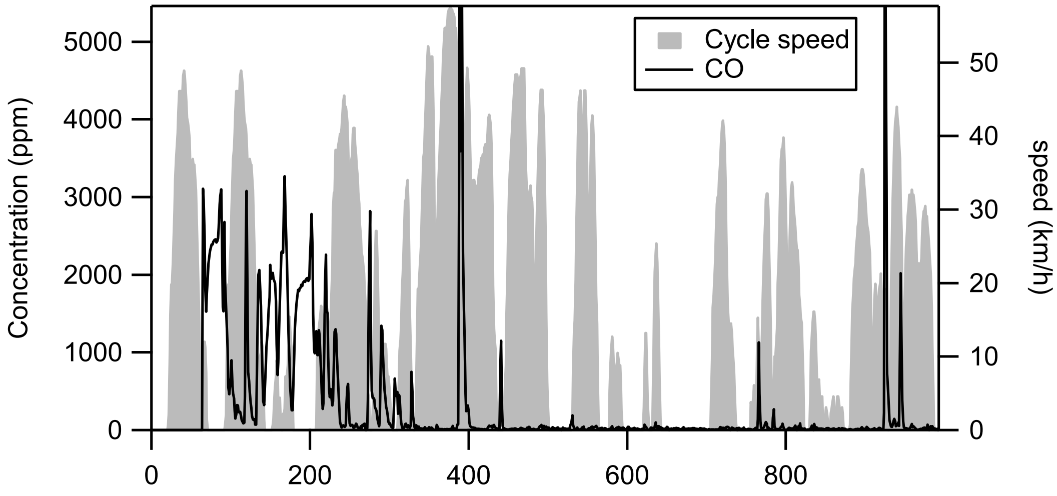

Figure S4. CO variations (ppm) during a diesel cold urban cycle.

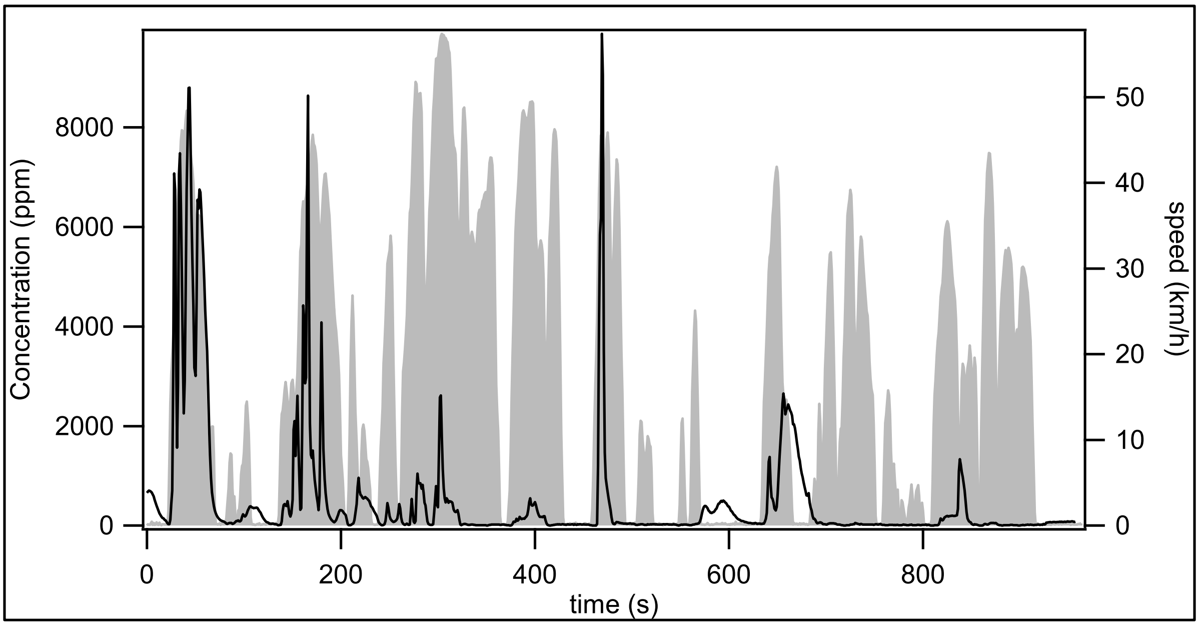

Figure S5. CO variations (ppm) during a GDI cold urban cycle. The black line represents CO concentration, the grey zone represent the cycle speed in km/h.

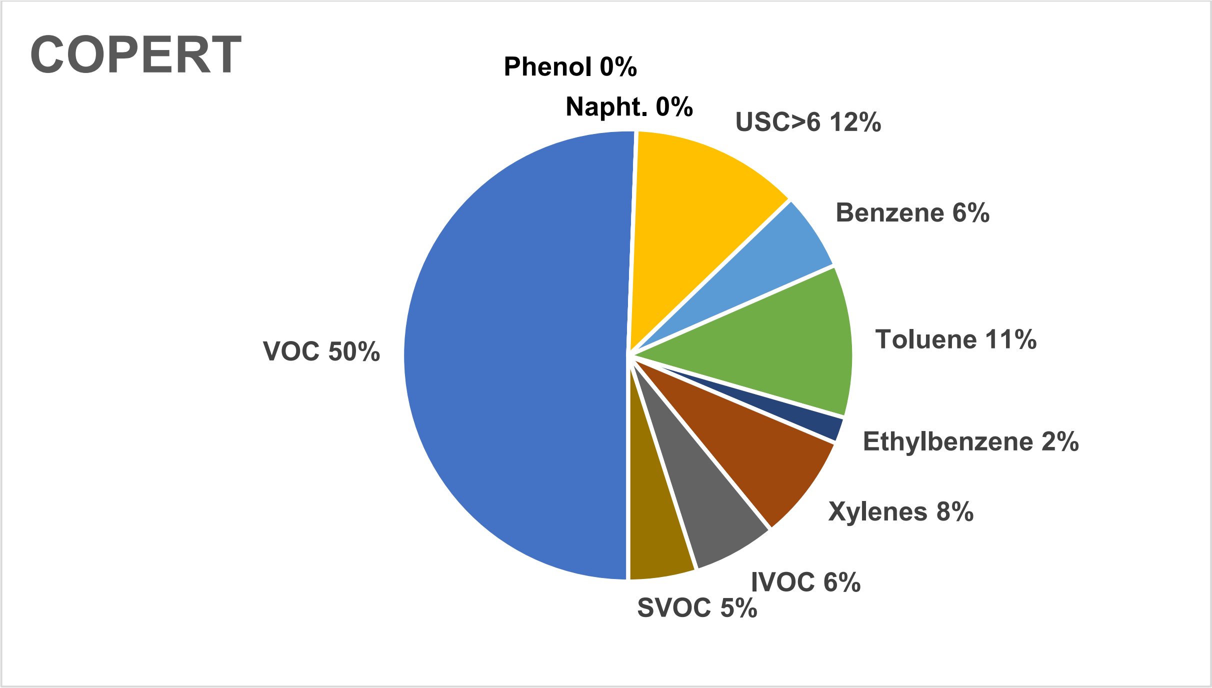

Figure S6. Complete NMVOC speciation from COPERT emission inventory for Euro1-6 gasoline passenger cars.

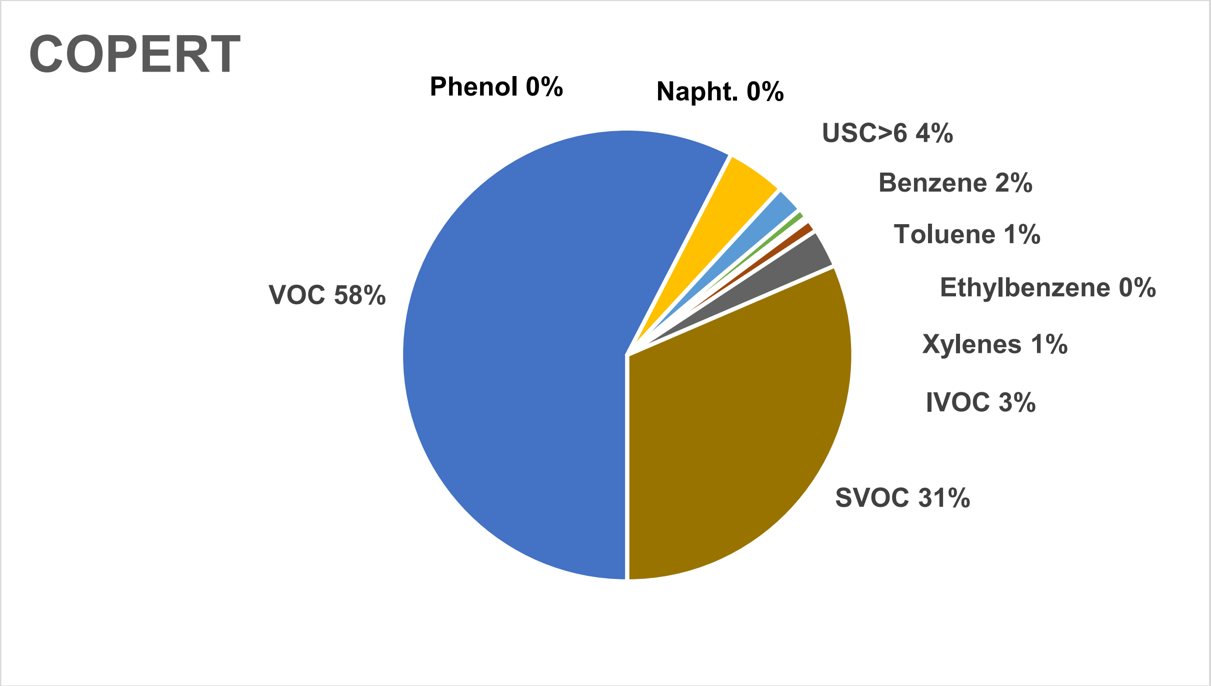

Figure S7. Complete NMVOC speciation from COPERT emission inventory for Euro1-6 diesel IDI and DI cars.
